# Targeting the E1 ubiquitin-activating enzyme (UBA1) improves elexacaftor/tezacaftor/ivacaftor efficacy towards F508del and rare misfolded CFTR mutants

**DOI:** 10.1007/s00018-022-04215-3

**Published:** 2022-03-16

**Authors:** Christian Borgo, Claudio D’Amore, Valeria Capurro, Valeria Tomati, Elvira Sondo, Federico Cresta, Carlo Castellani, Nicoletta Pedemonte, Mauro Salvi

**Affiliations:** 1 Department of Biomedical Sciences, University of Padova, Via Ugo Bassi 58/B, 35131 Padova, Italy; 2 UOC Genetica Medica, IRCCS Istituto Giannina Gaslini, Via Gerolamo Gaslini 5, 16147 Genova, Italy; 3 Centro Fibrosi Cistica, IRCCS Istituto Giannina Gaslini, Genova, Italy

**Keywords:** Genetic disease, Protein homeostasis, mln7243, Protein degradation, Chloride channel, Channelopathies

## Abstract

The advent of Trikafta (Kaftrio in Europe) (a triple-combination therapy based on two correctors—elexacaftor/tezacaftor—and the potentiator ivacaftor) has represented a revolution for the treatment of patients with cystic fibrosis (CF) carrying the most common misfolding mutation, F508del-CFTR. This therapy has proved to be of great efficacy in people homozygous for F508del-CFTR and is also useful in individuals with a single F508del allele. Nevertheless, the efficacy of this therapy needs to be improved, especially in light of the extent of its use in patients with rare class II CFTR mutations. Using CFBE41o- cells expressing F508del-CFTR, we provide mechanistic evidence that targeting the E1 ubiquitin-activating enzyme (UBA1) by TAK-243, a small molecule in clinical trials for other diseases, boosts the rescue of F508del-CFTR induced by CFTR correctors. Moreover, TAK-243 significantly increases the F508del-CFTR short-circuit current induced by elexacaftor/tezacaftor/ivacaftor in differentiated human primary airway epithelial cells, a gold standard for the pre-clinical evaluation of patients’ responsiveness to pharmacological treatments. This new combinatory approach also leads to an improvement in CFTR conductance on cells expressing other rare CF-causing mutations, including N1303K, for which Trikafta is not approved. These findings show that Trikafta therapy can be improved by the addition of a drug targeting the misfolding detection machinery at the beginning of the ubiquitination cascade and may pave the way for an extension of Trikafta to low/non-responding rare misfolded CFTR mutants.

**Supplementary Information:**

The online version contains supplementary material available at 10.1007/s00018-022-04215-3.

## Introduction

Cystic Fibrosis (CF) is a monogenic recessive disease caused by mutations in the cystic fibrosis transmembrane conductance (CFTR) gene. This gene encodes an ion channel mediating chloride and bicarbonate transport across the luminal surface of epithelial cells in multiple organs. CF is therefore a multiorgan disease, but the most serious consequence is the degeneration of lung function [1]. The mutations causing CF are classified based on the molecular consequences on CFTR protein: defects in protein expression (class I), impairment of protein trafficking/maturation (class II), gating defects (class III), conduction defects (class IV), insufficient protein (class IV), or defects in stability at the apical membrane (class VI) [2]. The most common worldwide mutation F508del is the prototype of class II mutants, encoding a protein with a folding defect entrapped in ER/Golgi compartments and subjected to premature degradation. However, F508del can also be included in class III, exhibiting a channel-gating defect when corrected, and in class VI, showing an accelerated turnover at the plasma membrane [2], suggesting that a multi-targeting approach would be necessary to treat this mutant. In 2015, Orkambi® (Vertex Pharmaceuticals, Cambridge, MA), a combined treatment with the corrector VX-809 (lumacaftor) and the potentiator VX-770 (ivacaftor), became the first drug treatment approved for people homozygous for F508del. This was followed in 2018 by Syndeko, in which the corrector VX-809 was substituted by the second-generation and better-tolerated corrector VX-661 (tezacaftor). However, the effectiveness of Orkambi® therapy has proved to be modest [3]. It was in 2019, with FDA approval of the triple-combination treatment Trikafta™ (Vertex Pharmaceuticals, Cambridge, MA), consisting of the two correctors VX-661 (tezacaftor) and VX-445 (elexacaftor) plus the potentiator VX-770, that F508del-CFTR therapy achieved results that until then could not have been expected. The importance of this new therapy should be ascribed not only to the very promising pre-clinical and clinical results but also to its wide coverage, as this treatment can be administered in patients with at least one F508del allele (70–90% of the cystic fibrosis population according to specific geographic distribution) [4]. However, a further improvement in F508del-CFTR rescue is still possible [5, 6]. Moreover, the therapeutic response varies between individuals and there is a significant group of CF patients in whom the response to the treatment is worse, and a minority that are forced to discontinue the therapy due to the side effects [7]. In terms of its efficacy on other mutants displaying defective maturation and trafficking to the plasma membrane, pre-clinical results on primary cells show a highly variable response to Trikafta treatment, and it is more effective in improving CFTR-mediated current with some mutants such as M1101K and less with others, including N1303K [8, 9], the most frequent class II mutant after F508del. The FDA recently agreed to extend the use of Trikafta to almost 200 different mutations which showed an increase in chloride transport to at least 10% of normal over baseline when expressed in Fisher rat thyroid cells and treated with the VX-445/VX-661/VX-770 combination [10]. It is worth noting that N1303K, as well as many other class II CFTR mutations, has not been included in this list.

As discussed above, CFTR class II mutants present a misfolded structure that is targeted by the cell quality control mechanism. As soon it was determined that the main mechanism of CFTR degradation is via polyubiquitination and proteasomal degradation [11], attempted inhibition of the proteasome was considered as a potential therapeutic option, although this approach was soon abandoned as it proved to be ineffective [11]. Therefore, the research shifted to anticipating the ubiquitination of the protein. Protein ubiquitination is based on a three-step mechanism involving E1, E2, and E3 enzymes. Ubiquitin-activating enzymes (E1) (ubiquitin-activating enzyme (UBA1) and ubiquitin-like modifier-activating enzyme-6 (UBA6)) catalyze ubiquitin “activation”, which in turn is transferred from E1 to a cysteinyl residue in the second class of proteins called ubiquitin-conjugating enzymes (E2) (⁓40). Transfer of ubiquitin to substrate proteins typically requires a third activity called E3 or ligase (⁓1000). Most of the CF research has focused on the identification of the E3 enzymes involved in controlling CFTR stability. Several E3 have been identified as having a role in promoting F508del-CFTR ubiquitination, including MARCH2 [12], RNF185 [13], gp78 [14], CHIP [15], RNF4 [16], RNF5 [17], RFFL [18], and RNF19B [19]. Even though targeting these E3 ubiquitin ligases increases the amount of F508del-CFTR protein and improves protein maturation induced by correctors, their effects are below expectations, suggesting that CFTR is under the control of multiple and possibly promiscuous ubiquitin ligases. It has recently been hypothesized that inhibiting E1 enzymes could be a more effective approach in preventing protein ubiquitination and increasing the amount of the protein suitable for correction [20]. In this regard, PYR-41, an E1 inhibitor, increases band B in HEK and CFBE41o- cells and, when used in combination with C4 corrector, also increases the matured band C [20]. Despite the potential importance of this approach, the specificity of PYR-41 has been seriously questioned. Indeed, it has been shown that this compound has a similar or greater inhibitory efficacy against several deubiquitinases and also exhibits inhibitory efficacy on some protein kinases [21]. In a follow-up study, Brodsky’s group has investigated the effects of a variety of structural analogs of PYR-41 on F508del and selected a compound with reduced toxicity and improved efficacy to facilitate F508del rescue induced by VX-809 in HEK, FRT, and CFBE cells [22].

Here, we are using a first-in-class specific E1 enzyme inhibitor: TAK-243 (also known as MLN7243) [23], which has not yet been assayed to our knowledge in CF. The primary target of TAK-243 is UBA1, the main E1 enzyme regulating the ubiquitin conjugation cascade [23]. In mammals, UBA1 is responsible for charging more than 99% of cellular ubiquitin, catalyzing the charge of almost all E2 ubiquitin-activating enzymes, except for the E2 USE1, whose charging is catalyzed by UBA6 [24]. TAK-243 also inhibits, with less efficacy, UBA6 (a ubiquitin- and Fat10-activating enzyme), NEDD8 E1*-*Activating Enzyme (NAE), and the SUMO-activating enzyme (SAE) [23]. TAK-243 was developed for cancer treatment and entered into clinical trials for treating patients with advanced solid tumors (NCT02045095) and with leukemia (NCT03816319).

Here, we show that TAK-243 stabilizes the F508del-CFTR protein but also that this is not a sufficient condition to induce F508del-CFTR functional recovery. Nevertheless, protein stabilization induced by TAK-243 substantially improves the efficacy of VX-445 and VX-661 in inducing F508del-CFTR rescue in both immortalized cells and patient airway epithelial cells. This new combinatory approach has also been assayed on different rare mutations displaying defective CFTR maturation, and although it cannot be generalized for all class II mutants, it also improves CFTR conductance on cells expressing severe mutants, including N1303K. As mentioned above, the two correctors have limited efficacy on N1303K mutations.

To summarise, our results show that the current therapy based on correcting trafficking (VX-445 and VX-661) and gating defects (VX-770) could be improved by the addition of a molecule specifically targeting the misfolding detection machinery at the beginning of the ubiquitination cascade.

## Materials and methods

Materials: VX-445, VX-661, VLX1570, Pevonedistat, and TAK-243 were purchased from MedChemExpress. MG-132 was purchased from Merck. Anti-CFTR antibodies (#596 and #570) were obtained by the Cystic Fibrosis Foundation Therapeutics, anti-CFTR (Clone 24–1) for immunoprecipitation was purchased from bio-techne. Anti-α-tubulin (T5168), anti-β-actin (A5441), anti-UBA6 (HPA037001), and anti-HA antibodies (H9658) were purchased from Merck. Anti-calnexin (sc-46669), anti-ubiquitin (sc-8017), anti-UBA1 (sc-53555), anti-NEDD8 (sc-373741), anti-HSP90 (sc-7947), anti-GRP78 (sc-13539), anti-HSP70 (sc-7298), and anti-KCa3.1 (sc-365265) were from Santa Cruz Biotechnology. Anti-pS51-eiF2α (ab32157) and anti-eiF2α (ab5369) were purchased from Abcam. HRP-conjugated secondary antibodies, anti-mouse, and anti-rabbit were from PerkinElmer. Alexa Fluor-488 and Alexa Fluor-594 secondary antibodies were purchased from Thermofisher. siRNA oligos were obtained from Thermofisher.

Cell culture: CFBE41o- expressing an endogenous level of F508del-CFTR or stably overexpressing F508del-CFTR and YFP-H148Q/I152L (a kind gift from Prof. L.J.V. Galietta, TIGEM, Italy) were cultured as in [25].

To perform a TAK-243 chronic treatment, F508del-CFTR overexpressing CFBE41o- cells were cultured in the presence of DMSO or 10 nM TAK-243 by replacing the culture media and adding the drug every three days for at least 1 month.

Isolation, culture, and differentiation of primary airway epithelial cells were performed as previously described [26] with some modifications. Bronchial epithelial cells were obtained from mainstem human bronchi derived from CF individuals undergoing lung transplants. Epithelial cells were detached by overnight treatment of bronchi with protease XIV. For the present study, cells were obtained from two F508del homozygous CF patients (HBE93 and HBE111). Nasal epithelial cells were obtained through a nasal brushing of both nostrils. For the present study, cells were obtained from rom six CF patients having different genotypes: HNE011 (N1303K/1717-1G > A), HNE084 (N1303K/N1303K), HNE006 (N1303K/R1066C), HNE001 (G542X/R1066C), HNE016 (G542X/R334W), and HNE020 (R347P/R347P). Airway epithelial cells were cultured in a serum-free medium (LHC9 mixed with RPMI 1640, 1: 1) containing various hormones and supplements, which favors cell number expansion, including (for nasal cells) ROCK and SMAD inhibitors (DMH-1, A-83–01, and Y-27632 compounds, [27]). The culture medium contained in the first days a mixture of different antibiotics (including colistin, piperacillin, and tazobactam) to eradicate bacterial contamination.

To obtain differentiated airway epithelia, bronchial or nasal cells were seeded at high density (500.000 cells/cm^2^) on porous membranes (Snapwell inserts, Corning, code 3801). After 24 h, the serum-free medium was removed from both sides and, on the basolateral side only, replaced with Pneumacult ALI medium (StemCell Technologies), and differentiation of cells (up to 16–18 days) was performed in air–liquid interface (ALI) condition.

MTT cell viability assay: CFBE41o- cells were plated on 96-well plates (20,000 cells per well). After 24 h, cells were treated with different concentrations of test compounds or vehicle alone (DMSO). The following day, the 3-(4,5-dimethylthiazol-2-yl)-2,5-diphenyltetrazolium bromide (MTT) substrate was added to the medium at a final concentration of 0.5 mg/ml and incubated for 1 h at 37 °C. Formazan crystals (proportional to the number of viable cells) are then dissolved by the addition of 20 μl of a pH 4.7 solution containing 20% (w:v) SDS, 50% (v:v) *N*,*N*-dimethylformamide, 2% (v:v) acetic acid, and 25 mM HCl, and quantified by recording the absorbance at 570 nm using an Infinite M200 PRO plate reader (TECAN, Life Sciences). All results were reported as the percentage of vehicle-treated cells.

Cell lysis and Western Blotting: Cells were washed twice with PBS and harvested with ice-cold lysis buffer containing 50 mM Tris–HCl (pH 7.5), 150 mM NaCl, 1% NP-40 (v/v), supplemented with protease inhibitor cocktail (Calbiochem) and phosphatases inhibitors cocktail 2 and 3 (Sigma-Merck). Cell lysates were centrifuged at 10000*g* for 10 min at 4 °C and protein concentration was determined by the Bradford method. 20 µg of total protein extracts were loaded on SDS-PAGE, blotted on Immobilon-P membranes (Millipore), processed by western blot with the indicated antibodies. Immunostained bands were detected by chemiluminescence on ImageQuant LAS 500 (GE Healthcare Life Sciences) and quantified with Carestream Molecular Imaging software (Carestream, Rochester, NY, USA).

Cell transfection and RNA interference: Plasmid transfections in CFBE41o- cells were performed as in [25]. For RNA interference, CFBE41o- overexpressing F508del-CFTR were transiently transfected for 48 h with 5 nM of UBA1 (#s599 and #s600) or UBA6 (#s30515 and #s30516) siRNA or scrambled siRNA (#4390843) (Silencer Select siRNAs, Thermo Fisher Scientific) with RNAiMAX (ThermoFisher Scientific), according to the manufacturer’s instructions.

Immunoprecipitation: Proteins (300 μg in 150 μl) from lysates of CFBE41o- overexpressing F508del-CFTR treated for 24 h with TAK-243 were immunoprecipitated overnight with 1 μg of anti-CFTR (clone 24–1), followed by the addition of protein A-Sepharose for 30 min. The immunocomplexes, washed three times with 50 mM Tris–HCl, pH 7.5, were analyzed by western blotting with the indicated antibodies.

Biotinylation of cell surface proteins: CFBE41o- cells stably expressing F508del-CFTR were seeded on a 100-mm dishes and, the day after, treated for 24 h with vehicle (DMSO) or with TAK-243 (200 nM), or with correctors (10 μM VX-661 + 3 μM VX-445), or with the combination of correctors and TAK-243. After treatment cells were washed twice with ice-cold PBS and exposed to sulfo-NHS-SS-biotin for 25 min on a shaker in a cold room, rinsed three times with PBS, and incubated 15 min with 50 mM NH_4_Cl in PBS (quenching buffer). Cells were washed twice with PBS and lysed as described above by scraping. Cell lysates were centrifuged at 10000*g* for 10 min at 4 °C and protein concentration in the supernatant was determined by the Bradford method. For each sample, 400 μg of lysate was incubated with high-capacity Streptavidin Agarose (ThermoFisher) for 3 h at 4 °C. After centrifugation, the supernatant was removed, and the beads were washed once with lysis buffer, twice with the following buffer: 150 mM NaCl, 20 mM Tris–HCl, pH 8, 5 mM EDTA, 1% Triton X-100, 0.2% BSA, once with the following buffer: 150 mM NaCl, 20 mM Tris–HCl, pH 8, 5 mM EDTA, 0.5% Triton X-100, and once with 50 mM Tris–HCl, pH 8. Biotinylated proteins were eluted from the resin with Laemli buffer supplemented with 100 mM DTT, loaded in SDS-PAGE, and analyzed by western blotting.

CFTR stability: F508del-CFTR overexpressing CFBE41o- cells were treated with DMSO or TAK-243 in combination or not with correctors (10 μM VX-661 + 3 μM VX-445) for 24 h. Subsequently, protein synthesis was inhibited by adding 100 µg/ml cycloheximide (CHX) to the culture medium, and then, cells were harvested at 0, 2, 4, and 8 h post-CHX treatment. Cell lysates were subjected to SDS-PAGE followed by western blotting to evaluate CFTR expression, as described previously.

Immunofluorescence: CFBE41o- cells stably overexpressing F508del-CFTR were seeded on a 12 mm glass coverslip, placed in a well of a 24-well plate, and let adhere for 24 h. Next, cells were left untreated or stimulated with TAK-243 200 nM for 18 h. Cells were fixed with 10% formalin (15 min at 4 °C), permeabilized with 0,1% TritonX-100 in PBS (10 min at 4 °C), and incubated with anti-CFTR antibody (#570, 1:50 in 3% BSA-PBS, 90 min at RT). Following PBS washes, cells were fluorescently labeled with Alexa Fluor-488 secondary antibody (1:500 in 3% BSA-PBS, 30 min at RT), and coverslips were mounted on microscope slides using Mowiol 40–88 (Sigma-Aldrich). Images were acquired under an Leica-TCS SP5 confocal microscope equipped with an Leica HCX PL APO 63 × 1.4 oil immersion objective.

In another experimental setting, for the determination of both plasma membrane and total CFTR, CFBE41o- cells were transfected with a pNUT plasmid encoding for a 3HA-tagged F508del-CFTR(a generous gift from prof. G. Lukacs, McGill University, Montreal, Canada). 24 h post-transfection, cells were left untreated or stimulated with DIK, 200 nM TAK-243, or with the combination of TAK-243 and CFTR correctors for a further 18 h. For the staining of CFTR at the plasma membrane, living cells were blocked with 3% BSA-PBS and incubated with anti-HA antibody (H9658, Sigma-Aldrich; 1:200 in 3% BSA-PBS, 1 h at 4 °C). Cells were then fixed with 10% formalin (15 min at 4 °C) and incubated with Alexa Fluor-488 secondary antibody (1:500 in 3% BSA-PBS, 30 min at RT). Next, cells were fixed again (10% formalin, 10 min at 4 °C), permeabilized with 0.1% TritonX-100 in PBS (10 min at 4 °C), and incubated again with anti-HA primary antibody (1:200 in 3% BSA-PBS, 90 min at RT). Total CFTR was stained using Alexa Fluor-594 secondary antibody (1:500 in 3% BSA-PBS, 30 min at RT). Coverslips were mounted on microscope slides and analyzed by confocal microscopy.

Fluorescent images were processed using the Fiji software.

Nuclear staining of CFBE41o- cells has been performed seeding cells on a 12 mm glass coverslip placed in a well of a 24-well plate, and letting adhere for 24 h. The cells were washed twice with cold PBS, fixed with 10% formalin (15 min at 4 °C), and then stained with Hoechst 33342 (1 μg/ml) for 15 min in the dark. The stained cells were observed with an epifluorescence microscope (×40 magnification, Zeiss Axio Observer).

RT-PCR: CFBE41o- cells stably expressing the F508del-CFTR were seeded at 3 × 10^5^cells/well in a 6-well plate and let adhere for 24 h. Next cells were left untreated or stimulated with TAK-243 (200 nM) for 18 h. Total mRNA was isolated with “Total RNA Purification kit” (Norgen, Biotek Corp.) and reverse-transcribed using SuperScript III reverse transcriptase (Thermo Fisher). For quantitative RT-PCR, 5 ng of template was dissolved in a 20 µl solution containing forward and reverse primers (200 nM each) and 10 µl of SensiFAST SYBR No-ROX Mix, 2x (Bioline). All the reactions were performed in triplicate on a Real-Time PCR Cycler Rotor-Gene 3000 (Corbett Research); the thermal cycling conditions were as follows: 3 min at 95 °C, 40 cycles of 95 °C 10 s, 58 °C 20 s and 72 °C 30 s. The relative mRNA expression was calculated and expressed as 2^−ΔΔCt^. Primers were as follows: h18S AAACGGCTACCACATCCAAG and CCTCCAATGGATCCTCGTTA; hCFTR CTGGGCTAGGGAGAATGATG and GCCTTCCGAGTCAGTTTCAG.

Thermoaggregation assay: Thermoaggregation assays were performed as previously described [28] with some modifications. Briefly, cells were treated with TAK-243 (200 nM) or with VX-809 (3 µM), or with the vehicle (DMSO) as control. After 24 h, cells were lysed in 50 mM Tris–HCl (pH 7.5), 150 mM NaCl, 1% NP-40 (v/v), supplemented with protease inhibitor cocktail (Calbiochem) and phosphatases inhibitors cocktail 2 and 3 (Sigma-Merck) on ice, and centrifuged at 15000*g* for 15 min at 4 °C. Equal amounts of lysates (50 μg) were incubated to 0°, 20°, 30°, 40°, and 50° for 15 min. Macromolecular aggregates were eliminated by centrifugation at 15,000*g* for 15 min at 4 °C and the soluble F508del-CFTR in the supernatant was measured by western blotting.

CFTR mutagenesis: CFTR expression plasmid was mutated with the QuickChange site-directed mutagenesis kit (Agilent). All plasmids were sequenced to confirm mutagenesis.

HS-YFP assay: CFTR activity has been measured using CFBE41o- cells stably expressing both the F508del-CFTR and the halide sensitive YFP (H148Q/I152L), according to [29] with minor modifications. Briefly, cells were seeded on 96-well black microplates and let to adhere for 24 h. Cells were treated with indicated compounds for a further 18 h, eight replicate wells for each condition. On the day of the experiment, cells were washed with PBS (137 mM NaCl, 2.7 mM KCl, 8.1 mM Na_2_HPO_4_, 1.5 mM KH_2_PO_4_, 1 mM CaCl_2_, and 0.5 mM MgCl_2_) and incubated with 20 µM forskolin and 50 µM genistein in PBS, to fully stimulate F508del-CFTR-CFTR, for 25 min at 37 °C. Next, plates were transferred to an EnVision plate reader (Perkin Elmer) to determine CFTR activity: YFP fluorescence (Ex:485/14; Em:535/30) was continuously measured for 15 s, 2 s before and 13 s after the injection of an iodide-enriched solution (185 mM NaI, 2.7 mM KCl, 8.1 mM Na_2_HPO_4_, 1.5 mM KH_2_PO_4_, 1 mM CaCl_2_, and 0.5 mM MgCl_2_); the final I^−^ concentration was 100 mM.

To determine I^−^influx rate and CFTR activity, background fluorescence was subtracted and fluorescence was normalized to the initial value. Background fluorescence has been measured before the beginning of the assay. In the same plate used for the assay, background fluorescence has been continuously measured for 15 s in at least 6 wells in which cells have not been seeded but filled with the same buffers used in the experiment (PBS and iodide-enriched solution).

The final 12 s of the data recording for each well was fitted with an exponential function to calculate the initial slope (d*F*/d*t*).

Cell cycle and DNA content analysis: CFBE41o- control and treated cells were grown to approximately 50–60% confluence. 1 × 10^6^ cells were washed with PBS, fixed with 70% (v/v) cold ethanol overnight at 4 °C, centrifuged (300 g for 8 min), and washed in PBS. Cells were stained with Propidium Iodide (PI) (50 µg/ml), in the presence of RNAse A (0.5 mg/ml) (SIGMA) at 4 °C in the dark for 30 min. 50.000/sample cells were analyzed on FACS Canto analyzer (BD Biosciences, San Jose CA) and data processed by the BD FACSDiva software program (BD Biosciences).

Short-circuit current recordings: Snapwell inserts carrying differentiated bronchial or nasal epithelia were mounted in a vertical diffusion chamber resembling a Ussing chamber and having internal fluid circulation. Apical and basolateral hemichambers were filled with a symmetrical solution containing (in mM): 126 NaCl, 0.38 KH_2_PO_4_, 2.13 K_2_HPO_4_, 1 MgSO_4_, 1 CaCl_2_, 24 NaHCO_3_, and 10 glucose. Hemichambers were continuously bubbled with a gas mixture containing 5% CO_2_—95% air and the temperature of the solution was kept at 37 °C. The transepithelial voltage was short-circuited with a voltage-clamp (DVC-1000, World Precision Instruments; VCC MC8 Physiologic Instruments) connected to the apical and basolateral chambers via Ag/AgCl electrodes and agar bridges (1 M KCl in 1% agar). The offset between voltage electrodes and the fluid resistance was adjusted to compensate parameters before experiments. The short-circuit current was recorded by analog-to-digital conversion on a personal computer.

Statistical analysis: Results are presented as mean ± SD. For the experiments with *n* ≥ 6 with a normal distribution of the data, the statistical significance was calculated using unpaired Student’s *t* test (two-tailed) or, in case of multiple comparisons, by one-way ANOVA, followed by Bonferroni’s multiple comparison test. For experiments with *n* < 6, the statistical significance was calculated using the Mann–Whitney test or, in the case of multiple comparisons, by Kruskal–Wallis test, followed by Dunn’s multiple comparison test. Differences were considered statistically significant with *p* < 0.05. All statistical analyses and graphs were produced using Prism (GraphPad Software) software.

## Results

**Fig. 1:**
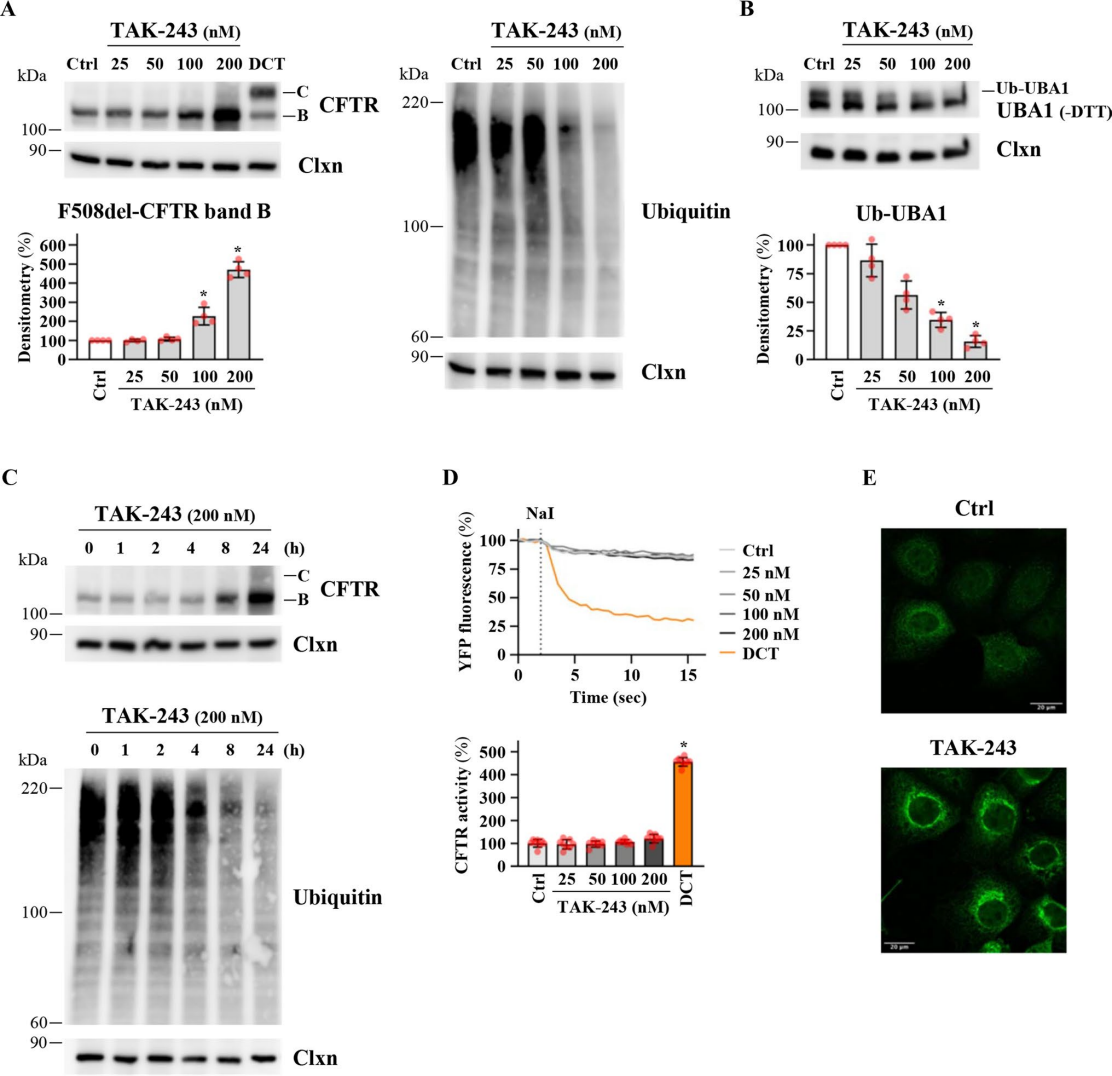
Effects of TAK-243 on F508del-CFTR expression and activity in CFBE41o- cells. **A** Immunoblot analysis of CFTR (upper left panel) or ubiquitinated proteins (right panel) in whole lysates from F508del-CFTR expressing CFBE41o- cells treated with DMSO (Ctrl) or TAK-243 (25, 50, 100, and 200 nM) or with double corrector treatment (DCT = 10 μM VX-661 + 3 μM VX-445) for 24 h. Calnexin (Clxn) was used as a loading control. The lower left panel shows the densitometric quantification of the immunostained F508del-CFTR band B in the experiment detailed in the upper left panel. The values for CFTR band B are expressed as a percentage of the control cells (means ± SD values, *n* = 4; *p < 0.05 vs Ctrl). **B** Cellular lysates were analyzed by western blot with anti-UBA1 antibody. Slower mobility of Ubiquitin–UBA1 thioester complexes (Ub–UBA1) was detected in the absence of reducing agent (-DTT). Calnexin (Clxn) was used as a loading control (upper panel). The lower panel shows the densitometric quantification of the immunostained Ub–UBA1 band. The values for Ub–UBA1 band are expressed as a percentage of the control cells (means ± SD values, *n* = 4; *p < 0.05 vs Ctrl). **C** Immunoblot analysis of CFTR (upper panel) or ubiquitinated proteins (lower panel) in whole lysates from F508del-CFTR expressing CFBE41o- cells treated with DMSO (Ctrl) or TAK-243 (200 nM) for the indicated times. Calnexin (Clxn) was used as loading control (*n* = 4). **D** HS-YFP assay in F508del-CFTR expressing CFBE41o- cells treated as indicated in panel **A**. Upper panel reports representative traces measuring YFP quenching (*n* = 8); the lower panel indicates the CFTR activity as a percentage of control (Ctrl) (means ± SD values, *n* = 8; *p < 0.05 vs Ctrl). **E** Confocal microphotographs of F508del-CFTR expressing CFBE41o- cells treated with DMSO (Ctrl) or TAK-243 (200 nM) for 24 h and immunostained for CFTR (upper and center panel); magnification 63x; scale bar 20 µm. The figure panel is representative of three independent experiments

TAK-243 increases the F508del band B amount without inducing channel rescue in CFBE41o-cells: CFBE41o- cells harboring the homozygous deletion of Phe-508 (F508del) in CFTR and overexpressing F508del-CFTR were treated with increasing doses of TAK-243 (25–200 nM, a range of concentrations not affecting cell proliferation, see Fig. S1A). The effect of TAK-243 on the expression of F508del-CFTR band B—the core-glycosylated ER-resident protein—and band C—the mature fully glycosylated protein—was assayed using western blotting. As a control, cells were treated with the combination of the correctors VX-445 and VX-661 (double-corrector treatment, DCT) used in the Trikafta therapy. The treatment with VX-445 and VX-661 induces a strong band C expression, proving their efficacy in F508del-CFTR rescue (Fig. 1A). TAK-243 induces a dose-dependent increase in F508del-CFTR band B expression without inducing any appreciable effect on band C (Fig. 1A). Dose-dependent inhibition of the protein ubiquitination process in parallel with the increase in band B expression confirms the efficacy of the inhibitor (Fig. 1A). To demonstrate that UBA1, the main molecular target of TAK-243, is inhibited at the concentrations used in these experiments, we revealed the UBA1-ubiquitin thioester species, running an SDS gel under non-reducing conditions. As part of the catalytic mechanism, active UBA1 forms reducing agent-sensitive thioesters with ubiquitin (UBA1-ubiquitin) that is transferred to an E2 enzyme in the ubiquitination cascade. Under non-reducing conditions, UBA1-ubiquitin thioester species with a slower migration rate can be distinguished from UBA1. Consistent with inhibition of its main target, TAK-243 decreased the level of DTT-sensitive UBA1-ubiquitin thioesters (Fig. 1B). The time-course analysis of 200 nM TAK-243 treatment shows that the effect on band B is observable after 8 h of incubation, in parallel with a general decrease in protein ubiquitination (Fig. 1C). In line with these results, we observed that TAK-243 treatment is ineffective in rescuing F508del-CFTR activity (Fig. 1D). These results were further confirmed by confocal analysis. Figure 1E shows a marked increase in F508del-CFTR signal after TAK-243 treatment confined to endogenous compartments. Collectively, these results show that TAK-243 has a considerable impact on band B stability/expression, but this is not a sufficient condition to induce F508del-CFTR recovery.

**Fig. 2:**
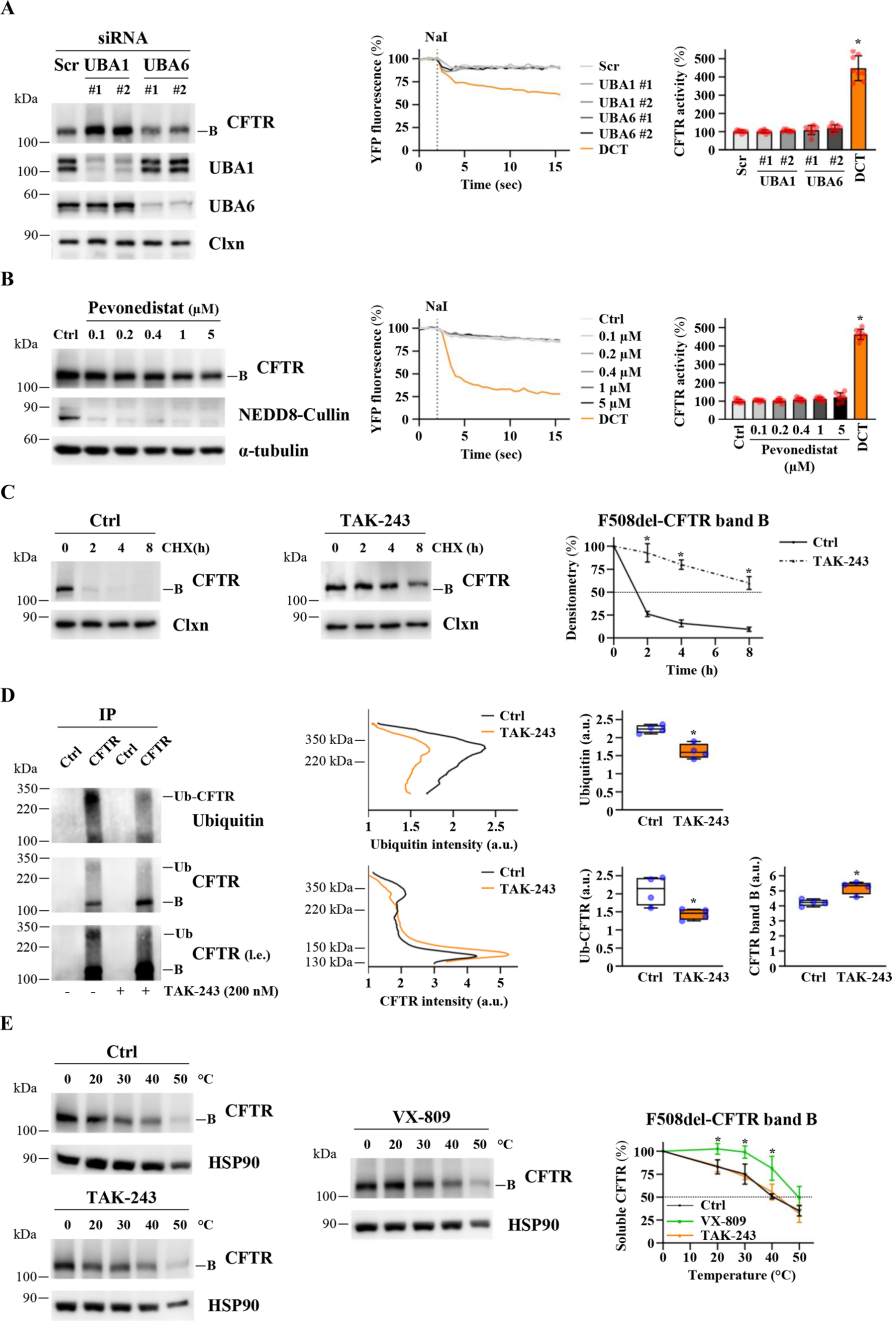
Identification of the molecular target of TAK-243 affecting F508del-CFTR cellular amount. **A** F508del-CFTR expressing CFBE41o- cells were transfected with non-specific siRNA (Scr), or UBA1 or UBA6-specific siRNAs for 48 h and lysed. Lysate proteins were analyzed by western blot with the indicated antibodies. Calnexin (Clxn) was used as loading control (*n* = 4) (left panel). Evaluation of F508del-CFTR activity was performed by HS-YFP assay in F508del-CFTR expressing CFBE41o- cells treated as indicated above. Centre panel exhibits representative traces measuring YFP quenching (*n* = 8); right panel shows the CFTR activity as a percentage of control (Scr) (means ± SD values, *n* = 8; *p < 0.05 vs Ctrl). **B** Immunoblot analysis of CFTR and NEDD8-Cullin in whole lysates from F508del-CFTR expressing CFBE41o- cells treated with DMSO (Ctrl) or increasing concentrations of Pevonedistat for 24 h. α-tubulin was used as loading control (*n* = 3) (left panel). Assessment of F508del-CFTR activity was carried out by HS-YFP assay in F508del-CFTR expressing CFBE41o- cells treated as indicated above or with double-corrector treatment (DCT = 10 μM VX-661 + 3 μM VX-445) for 24 h. Centre panel exhibits representative traces measuring YFP quenching (*n* = 8); right panel shows the CFTR activity as a percentage of control (Ctrl) (means ± SD values, *n* = 8; *p < 0.05 vs Ctrl). **C** F508del-CFTR expressing CFBE41o- cells were treated with DMSO (Ctrl) or TAK-243 (200 nM) for 24 h. Subsequently, protein synthesis was inhibited by adding 100 µg/ml cycloheximide (CHX) and cells were harvested after 2, 4, and 8 h. Protein lysates were analyzed by western blot with anti-CFTR antibody. Calnexin (Clxn) was used as a loading control. The rightmost panel represents the densitometric quantification of the immunostained bands of F508del-CFTR (band B) normalized by the value at time = 0 (means ± SD values, *n* = 4; *p < 0.05 vs the value of the same time-point of Ctrl). **D** F508del-CFTR expressing CFBE41o- cells were treated with DMSO or 200 nM TAK-243 and lysate after 24 h. 300 µg of lysate proteins were immunoprecipitated with a control antibody from the same class (Ctrl) or anti-CFTR (CFTR) antibody. The immunocomplexes were analyzed by western blot with the indicated antibodies (left panel). The figure panel is representative of four independent experiments. A long exposition of CFTR detection is also shown (i.e., long exposition). The central panels show representative density profiles of CFTR and ubiquitin in the CFTR-immunoprecipitated samples. Quantification of the density profiles was performed in the right panels by integrating the profile curves in the indicated intervals of molecular weight (Ubiquitin: 220–350 kDa; Ub-CFTR: 220–350 kDa; CFTR band B: 130–150 kDa) (means ± SD; *n* = 4; *p < 0.05 vs Ctrl). **E** Immunoblot analysis of CFTR in whole lysates derived from F508del-CFTR expressing CFBE41o- cells treated with DMSO (Ctrl, black) or TAK-243 (200 nM) (orange) or VX-809 (3 µM) (green), following heat-denaturation at indicated temperatures (left panels and central panel). Right panel reports the densitometric quantification of aggregation-resistant F508del-CFTR band B, normalized by HSP90 expression (means ± SD; *n* = 3; *p < 0.05 vs the value of the same temperature-point of Ctrl). CFTR melting temperature (mean ± SD): Ctrl = 41.96 ± 2.35 °C; TAK-243 = 42.64 ± 2.60 °C; VX-809 = 49.73 ± 3.47 °C

Inhibition of UBA1 is responsible for the TAK-243 effects on F508del-CFTR: As stated above, the primary target of TAK-243 is UBA1 (IC_50_ 1 ± 0.2 nM) and the secondary targets are UBA6 (IC_50_ 7 ± 3 nM), NAE (IC_50_ 28 ± 11 nM), and SAE (IC_50_ 850 ± 180 nM) [23]. UBA1, UBA6, and NAE could be affected by the concentrations used in this study. We performed the following experiments to determine whether one or more of the TAK-243 secondary targets could be involved in the regulation of F508del-CFTR band B expression/stability. First, we downregulated UBA1 and UBA6 enzymes using siRNA treatments. Figure 2A shows that the downregulation of UBA6 does not induce any effect on F508del-CFTR protein expression, while the downregulation of UBA1 induces a substantial increase in band B without inducing maturation of the channel (observable with TAK-243). According to the previous results, F508del-CFTR activity is unaffected by siRNA treatments targeting UBA6 or UBA1 (Fig. 2A).

To investigate a possible contribution of NAE in the observed effects, we used a new NAE inhibitor—Pevonedistat (MLN4924) [30]. Pevonedistat behaves as an indirect inhibitor of cullin RING ligase (CRL), the largest family of E3 ubiquitin ligases, which requires neddylation for its activity. The role of neddylation in regulating F508del-CFTR ubiquitination and degradation has recently been highlighted [31]. It is worth noting that NEDD8 silencing in different cellular models has been shown to decrease channel ubiquitination, resulting in a partial rescue of F508del-CFTR processing and function, an effect amplified by co-treatment with the corrector C18 [31]. Unexpectedly, our results show that Pevonedistat does not influence F508del-CFTR expression and activity in CFBE41o- cells (24 h treatment, Fig. 2B, and 48 h treatment, Fig. S1C). Notably, the compound was used at sublethal concentrations for the cells (Fig. S1B) and its efficacy was confirmed by the inhibition of cullin neddylation (NEDD8-Cullin) (Figs. 2B and S1C). Taken together, these results show that UBA1 inhibition is the main mechanism responsible for the TAK-243 effects on F508del-CFTR in CFBE41o- cells.

TAK-243 increases F508del-CFTR stability in CFBE41o- cells. To better investigate the TAK-243 mechanism of action, the stability of F508del-CFTR band B following 24 h treatment with TAK-243 was analyzed after cycloheximide (CHX) treatment. Figure 2C shows that band B stability substantial increases after TAK-243 treatment, while CFTR gene transcription is unaffected (Fig. S1D). Moreover, the immunoprecipitation of F508del-CFTR after TAK-243 treatment (Fig. S1E) shows a reduction in CFTR-ubiquitinated forms (Fig. 2D).

To shed light on a possible effect of TAK-243 on F508del-CFTR protein folding, we measured the temperature that induces the conversion of detergent-solubilized CFTR into SDS-resistant aggregates in the presence or absence of the compound [32]. CFBE41o- cells overexpressing F508del-CFTR were treated for 24 h with TAK-243 or with VX-809 as control and then lysed. Cell lysates were incubated at different temperatures (from 0° to 50 °C) and centrifuged, and the supernatant representing the aggregation-resistant CFTR protein was quantified using western blotting. As shown in Fig. 2E, as expected, the corrector VX-809 increases the resistance to thermoaggregation of CFTR, suggesting an improvement in protein folding. However, TAK-243 fails to do this, showing that the compound is not able to correct the structure of the protein. To summarise, our results show that TAK-243 increases the amount of F508del-CFTR protein in the cell by decreasing its ubiquitination via direct inhibition of the UBA1 enzyme, but the drug is not able to improve F508del-CFTR folding and maturation.

**Fig. 3:**
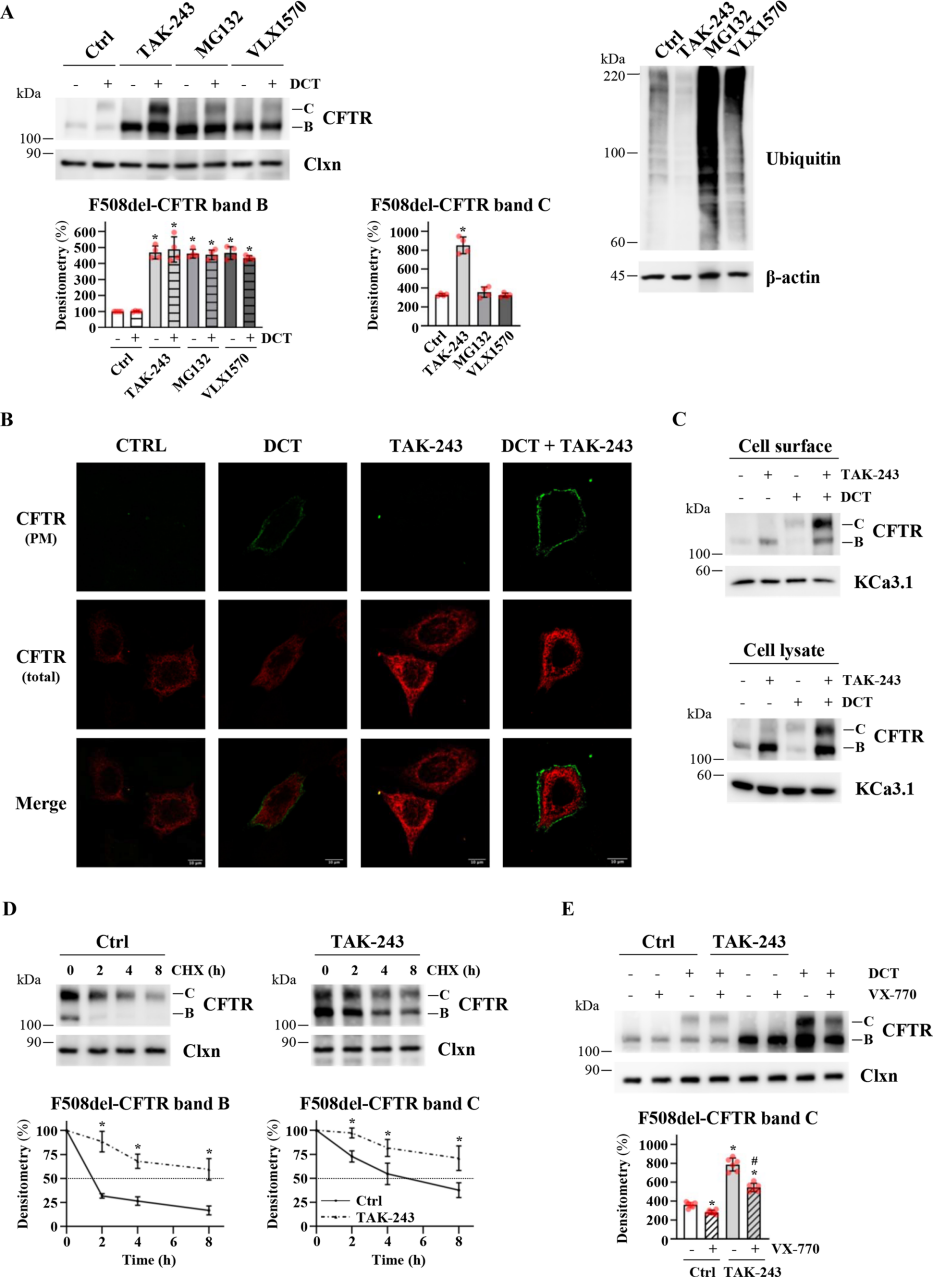
Comparison of the effect of double-corrector treatment with TAK-243 or proteasomal inhibitors on the F508del-CFTR recovery. **A** F508del-CFTR expressing CFBE41o- cells were treated with DMSO (Ctrl) or TAK-243 (200 nM) or MG132 (1 μM) or VLX1570 (250 nM) in combination or not with double-corrector treatment (DCT = 10 μM VX-661 + 3 μM VX-445) for 24 h. Subsequently, cells were lysed and analyzed by western blot. Calnexin (Clxn) and β-actin were used as a loading control. Lower panels show the densitometric quantification of the immunostained bands of F508del-CFTR band B (left) and band C (right) in the experiment detailed in the upper left panel. The values for CFTR band B are expressed as a percentage of the control cells not treated with DCT (means ± SD values, *n* = 4; *p < 0.05 vs Ctrl w/o DCT). CFTR band C values are reported for each experimental group (Ctrl, TAK-243, MG132, and VXL1570) as percentage of the cells not treated whit DCT (means ± SD values, *n* = 4; *p < 0.05 vs Ctrl). **B** Confocal microphotographs of F508del-CFTR expressing CFBE41o- cells treated with DMSO (Ctrl) or DCT (10 μM VX-661 + 3 μM VX-445) or TAK-243 (200 nM) or DCT + TAK-243 and immunostained for plasma membrane (PM) CFTR (green) or intracellular (total) CFTR (red) as described in Materials and methods section; magnification 63x; scale bar 10 µm. The figure panel is representative of three independent experiments. **C** Detection by cell surface biotinylation of CFTR expressed at the plasma membrane. Immunoblot detection of CFTR and control (KCa3.1) proteins in the cell surface (upper panel) and cell lysates (lower panel) from F508del-CFTR expressing CFBE41o- cells. **D** F508del-CFTR expressing CFBE41o- cells were treated with DMSO (Ctrl) or TAK-243 (200 nM) in combination with DCT (10 μM VX-661 + 3 μM VX-445) for 24 h. Subsequently, protein synthesis was inhibited by adding 100 µg/ml cycloheximide (CHX), and cells were harvested at the indicated times. Protein lysates were analyzed by western blot with an anti-CFTR antibody. Calnexin (Clxn) was used as a loading control (upper panels). Lower panels represent the densitometric quantification of the immunostained bands of F508del-CFTR band B (left) and band C (right) in the experiment detailed in upper panels, normalized by the value at time = 0 (means ± SD values, *n* = 4; *p < 0.05 *vs* the value of the same time-point of Ctrl). **E** F508del-CFTR expressing CFBE41o- cells were treated with DMSO (Ctrl) or TAK-243 (200 nM) in combination or not with DCT (10 μM VX-661 + 3 μM VX-445) in presence or not of VX-770 (1 μM) for 24 h. Afterward, cells were lysed and protein lysates were analyzed by western blot with an anti-CFTR antibody. Calnexin (Clxn) was used as a loading control (upper panel). The lower panel shows the densitometric quantification of the immunostained bands of F508del-CFTR band C in the experiment detailed in the upper panel. The values are expressed for Ctrl cells as percentage of untreated cells and for TAK-243 cells as percentage of the cells treated with the E1 inhibitor alone (means ± SD values, *n* = 6; *p < 0.05 *vs* Ctrl with DCT, #*p* < 0.05 *vs* Ctrl with DCT + VX-770)

TAK-243 boosts F508del-CFTR rescue induced by VX-445 and VX-661 in CFBE41o- cells. Even though TAK-243 fails to induce F508del rescue, we wondered whether the increased amount of F508del-CFTR band B induced by TAK-243 could be corrected using modulators. CFBE41o- cells overexpressing F508del-CFTR were treated with the two correctors VX-445 and VX-661 (double-corrector treatment, DCT) in the presence or absence of TAK-243. Figure 3A shows that the presence of TAK-243 increases band C expression by 250% compared to the correctors alone (Fig. 3A and Fig. S2A). The ratio between band C and band B does not increase in the presence of TAK-243 (band C/band B ratio 0.7–0.8 in the range of 100–200 nM), which confirms that this inhibitor is not able (as a single agent) to induce channel maturation and that the increase in band C observed using TAK-243 in combination with correctors is the result of a greater amount of F508del-CFTR available for correction. A similar effect is obtained by downregulating UBA1, the main target of TAK-243. Indeed, the effect of DCT treatment is greater in CFBE41o- cells treated with two different siRNAs targeting UBA1 compared with cells treated with scrambled siRNAs (Fig. S2B). Moreover, the YFP*-*halide assay detailed in Fig. S2C shows that the increased amount of mature F508del-CFTR in response to the combination of TAK-243 with correctors is accompanied by an increase in iodide influx at the plasma membrane. The localization of F508del-CFTR at the plasma membrane was also observed through confocal immunofluorescence using a plasmid expressing F508del-CFTR with an extracellular 3X-HA tag. Cells were incubated with an anti-HA antibody before cell permeabilization to label plasma membrane-resident F508del-CFTR (green) and stained with an anti-CFTR antibody after permeabilization to label total CFTR (red). Confocal immunofluorescence confirms that the addition of TAK-243 is not sufficient to induce F508del-CFTR trafficking to the plasma membrane (Fig. 3B). The green staining is observable only after DCT treatment and increases in the presence of TAK-243 (Fig. 3B). In parallel, we compared F508del-CFTR rescue at the plasma membrane using cell surface biotinylation. Figure 3C shows that TAK-243 substantially increases the amount of mutant CFTR at the plasma membrane induced by correctors.

Moreover, we compared the effect of TAK-243 with two other molecules able to prevent the proteasomal degradation of proteins but without preventing their ubiquitination: MG132 and VLX1570. Both molecules impair proteasome processing: MG132 by blocking the proteolytic activity of the 26S proteasome complex and VLX1570 by inhibiting proteasome deubiquitinase activity [33]. MG132 and VLX1570 added to CFBE41o- cells overexpressing F508del-CFTR have a TAK-243-like effect, increasing band B-only expression, but, in line with their different mechanisms of action, while MG132 and VLX1570 substantially increase total protein ubiquitination, TAK-243 has the opposite effect (Fig. 3A). Moreover, in contrast to TAK-243, this increase in F508del-CFTR protein cannot be corrected by VX-445/VX-661, as shown by western blotting (Fig. 3A) and channel activity (Fig. S2C). This result demonstrates that the functional recovery of F508del-CFTR cannot be achieved by blocking its degradation without preventing its ubiquitination.

We also investigated whether TAK-243 could improve the half-life of the mature form of the CFTR channel, the form usually expressed at the plasma membrane. For this purpose, we treated F508del-CFTR overexpressing CFBE41o- cells with correctors in the presence or absence of TAK-243. After 24 h treatment, cells were incubated with CHX to block protein synthesis and lysed at different times. Figure 3D shows that the presence of TAK-243 increases the stability of band C, confirming that prevention of F508del-CFTR ubiquitination has a double beneficial effect on the stability of both the immature (ER-resident) and the mature form (which reaches the plasma membrane) of the CFTR channel.

One limitation on the use of the potentiator VX-770 is that while, on the one hand, it increases channel activity, on the other, it accelerates plasma membrane turnover, decreasing the amount of protein at the cell surface [34]. We asked, therefore, whether TAK-243 could reduce this side effect of VX-770. Although the negative effect of VX-770 on CFTR plasma membrane stability is unaffected by TAK-243, in cells treated with TAK-243 and DCT, the F508del-CFTR band C amount after potentiator treatment remains significantly higher than in cells treated only with DCT and VX-770 (Fig. 3E).

**Fig. 4:**
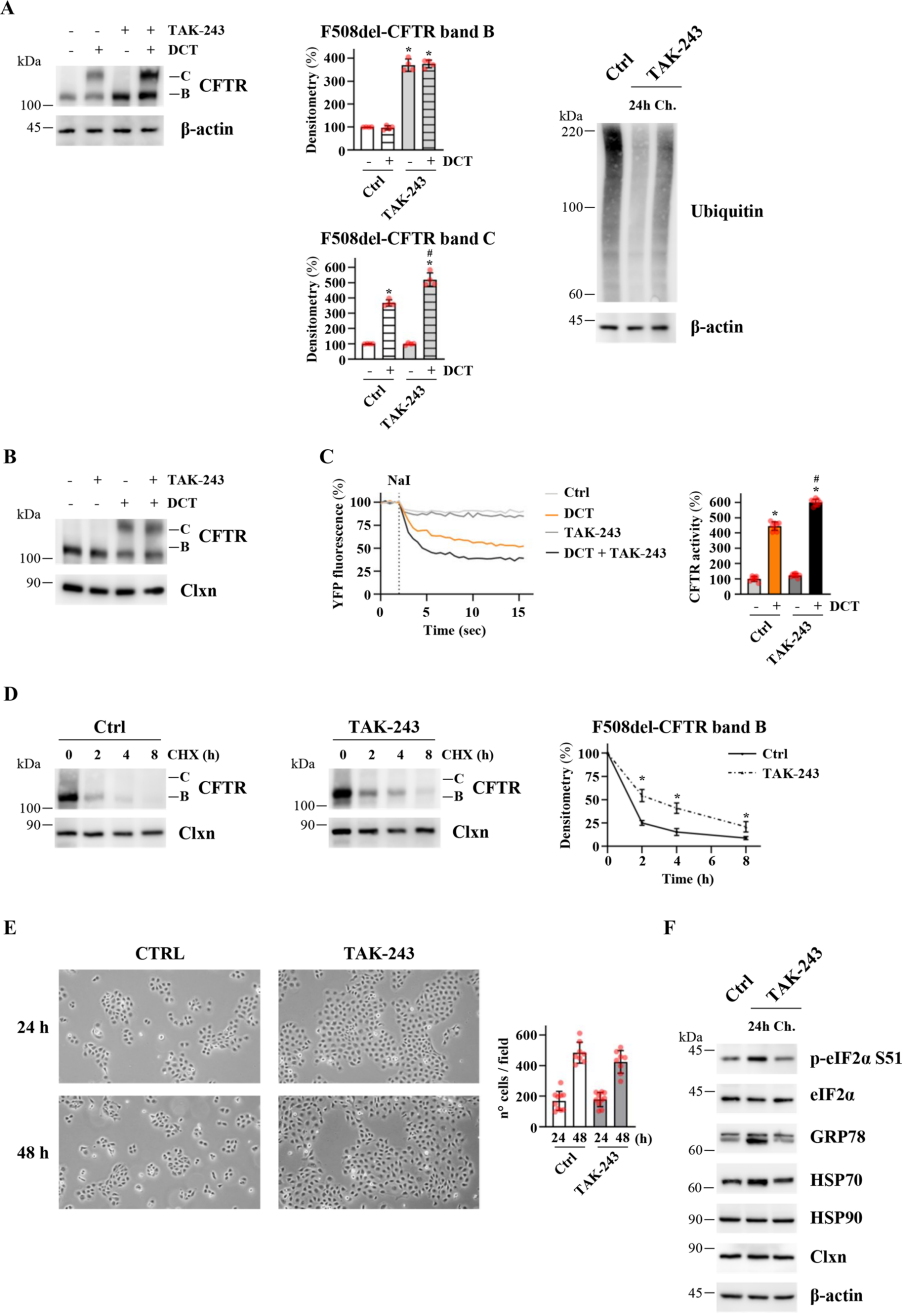
Effects of chronic treatment with low doses of TAK-243 on F508del-CFTR recovery in CFBE41o- cells. **A** Immunoblot analysis of CFTR in whole lysates from F508del-CFTR expressing CFBE41o- cells chronically treated (at least 1 months) with DMSO (Ctrl) or 10 nM TAK-243 and treated or not with double-corrector treatment (DCT = 10 μM VX-661 + 3 μM VX-445) for 24 h. β-actin was used as a loading control (left panel). Middle panels report the densitometric quantification of the immunostained bands of F508del-CFTR band B (upper) and band C (lower) in the experiment detailed in the left panel, expressed as a percentage of the control cells not treated with DCT (means ± SD values, *n* = 4; *p < 0.05 vs Ctrl w/o DCT, #*p* < 0.05 vs Ctrl with DCT). The right panel shows the immunoblot detection of ubiquitinated proteins in whole lysates from cells chronically treated (at least 1 month) with DMSO (Ctrl) or 10 nM TAK-243 (Ch.) or from cells treated with 200 nM TAK-243 for 24 h (24 h). β-actin was used as a loading control. The figure panel is representative of four independent experiments. **B** Immunoblot analysis of CFTR in whole lysates from F508del-CFTR expressing CFBE41o- cells treated with DMSO (Ctrl) or TAK-243 (10 nM) in combination ( +) or not (−) with double-corrector treatment (DCT = 10 μM VX-661 + 3 μM VX-445) for 24 h. **C** HS-YFP assay in F508del-CFTR expressing CFBE41o- cells treated as indicated in panel **A**. Left panel exhibits representative traces measuring YFP quenching (*n* = 4); the right panel shows the CFTR activity as a percentage of control cells not treated with DCT (Ctrl) (means ± SD values, *n* = 8; *p < 0.05 vs Ctrl w/o DCT, #*p* < 0.05 vs Ctrl with DCT). **D** DMSO (Ctrl) or TAK-243 (10 nM) chronically treated cells were treated with 100 µg/ml cycloheximide (CHX) and cells were harvested at the indicated times. Protein lysates were analyzed by western blot with an anti-CFTR antibody. Calnexin (Clxn) was used as a loading control. The right panel represents the densitometric quantification of the immunostained bands of F508del-CFTR (band B) in the experiments detailed in left and center panels, normalized by the value at time = 0 (means ± SD values, *n* = 4; *p < 0.05 vs the value of the same time-point of Ctrl). **E** Representative microphotograph of F508del-CFTR expressing CFBE41o- cells chronically treated (at least 1 months) with DMSO (Ctrl) or TAK-243 (10 nM) after seeding 24–48 h; magnification 5 × (left panel). The right panel shows the quantifications of cells number at the indicated time (means ± SD values, *n* = 8). The mean percent increase in the number of cells in the interval between 24 and 48 h is 276% and 249%, respectively **F** F508del-CFTR expressing CFBE41o- cells chronically treated (at least 1 month) with DMSO (Ctrl) or 10 nM TAK-243 (Ch.) or F508del-CFTR expressing CFBE41o- cells treated with 200 nM TAK-243 for 24 h (24 h) were lysed and protein lysates were analyzed by western blot with the indicated antibodies. β-actin was used as a loading control. The figure panel is representative of four independent experiments

TAK-243 chronic treatment of CFBE41o- cells does not affect cell viability. After having proven the efficacy of TAK-243 in combination with correctors in inducing F508del-CFTR rescue, we investigated whether the possible transfer of this molecule into clinical practice could be prevented by its potential toxicity. Indeed, TAK-243 inhibits the E1 enzyme catalyzing the first step in the pathway leading to the formation of ubiquitin–protein conjugates, and could therefore prevent a large proportion of ubiquitination processes in the cell. We wondered whether a partial inhibition of the E1 enzyme could be sufficient to improve the correction of CFTR class II mutants. We therefore administered chronic treatment (at least 1 month) to CFBE41o- cells overexpressing F508del-CFTR with a TAK-243 concentration 20 times lower than that used in the previous experiments (10 nM). The compound was added with each change of cell medium (every 3 days). The addition of correctors to 10 nM of cells treated chronically with TAK-243 shows greater band C expression compared with DCT alone (Fig. 4A), while the 24 h treatment of F508del-CFTR-overexpressing CFBE41o- cells with 10 nM of TAK-243 in combination with DCT does not improve the channel maturation induced by the correctors alone (Fig. 4B). Moreover, the increase in band C expression in chronically treated cells occurs in parallel with the increase in channel activity (Fig. 4C). The chronic treatment reduces the ubiquitination of total proteins (Fig. 4A) and F508del-CFTR (Fig. S3A). We also confirmed that F508del-CFTR protein stability is increased in cells treated chronically with TAK-243 compared to control cells (Fig. 4D).

Regarding the potential toxicity of this type of treatment, we observed that the TAK-243 chronic treatment (CFBE41o- cells treated for at least one month) has no effect on cell proliferation (Fig. 4E) and does not induce nuclear morphological alterations (nuclear fragmentations, chromatin condensation) (Fig. S3B). Moreover, the chronic low-dose treatment does not affect cell cycle progression and no modifications in DNA cell content are detected (Fig. S3C). It has been observed that TAK-243 cell treatments can induce ER-stress [23]. We assayed the ER-stress markers in both the 200 nM-24 h and 10 nM-chronic TAK-243 treatments (Fig. 4F, Fig. S3D). The 24 h-TAK-243 treatment induces some form of ER-stress induction, as shown by the increase in the expression of GRP78 and HSP70 and the phosphorylation extent of Ser51 eIF2α. However, a chronic low-dose treatment has no effect on the induction of ER-stress markers (Fig. 4F, Fig. S3D).

To summarise, our data on F508del-CFTR-overexpressing CFBE41o- cells suggest that TAK-243 in CFBE41o- cells improves the therapeutic potential of correctors on F508del-CFTR and that a chronic dose of TAK-243 effective in amplifying the correction induced by VX-445/VX-661 could be tolerated by CFBE41o- cells.

TAK-243 significantly increases F508del-CFTR conductance induced by elexacaftor/tezacaftor/ivacaftor in differentiated human primary airway epithelial cells homozygous for F508del-CFTR. Before the clinical benefit of a new therapeutic formulation can be predicted, its effect on suitable pre-clinical models should be verified, as most of the compounds that work on CFBE41o- cells often fail to work in more complex disease models [35–39].

**Fig. 5:**
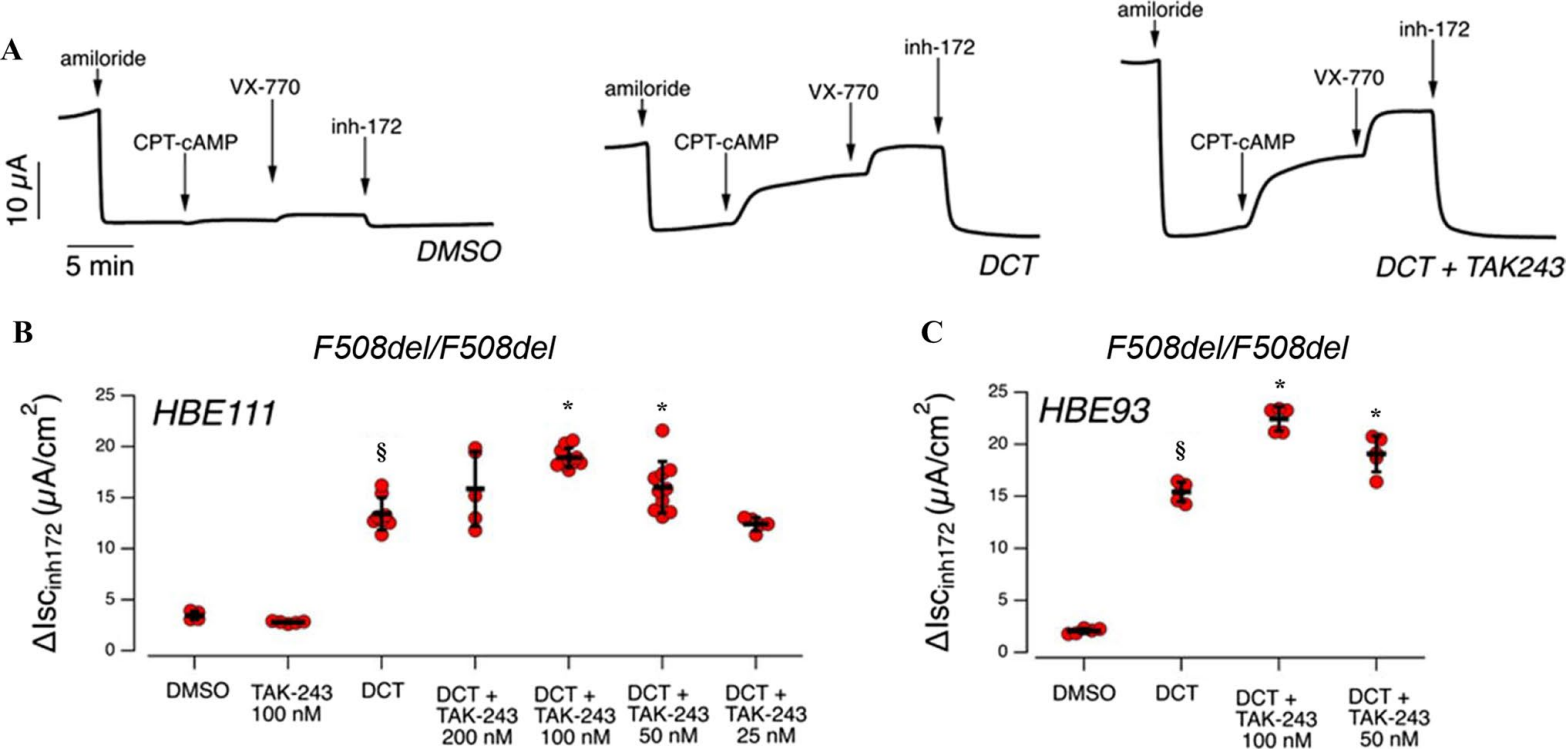
Functional evaluation of DCT and TAK-243 co-treatment on F508del/F508del human bronchial epithelial cells. **A** Representative traces of the effect of the vehicle alone (DMSO) or VX-661/VX-445 (10 µM/3 µM; DCT), or VX-661/VX-445 plus TAK-243 (100 nM), in F508del/F508del bronchial epithelial cells (HBE931) with the short-circuit current technique. **B** Summary of results obtained from short-circuit current recordings on F508del/F508del bronchial epithelial cells derived from two different CF patients (HBE93 and HBE111). Data reported are the amplitude of the current blocked by 10 µM inh-172. Symbols indicate statistical significance of DCT vs control (DMSO-treated): means ± SD values, ^§^*p* < 0.05; or of DCT plus TAK-243 vs DCT *p < 0.05

For this reason, we determined the ability of TAK-243 to improve the effect of VX-445/VX-661 correctors on well-differentiated primary cultures of human bronchial cells derived from two different F508del-CFTR homozygous patients using short-circuit current measurements. Bronchial epithelia differentiated under air–liquid conditions were treated for 24 h with TAK-243 alone or with VX-445/VX-661 in the presence or absence of different concentrations (in the 25–200 nM range) of TAK-243. The epithelia were then mounted in an Ussing chamber to measure CFTR-dependent Cl- secretion under short-circuit current conditions (Fig. 5). First, the epithelial sodium-channel ENaC was blocked using amiloride (10 µM), and cells were then stimulated using the non-hydrolyzable, membrane-permeable cAMP analog CPT-cAMP (100 µM) followed by the potentiator VX-770 (1 µM) to ensure maximum activation of F508del-CFTR (Fig. 5A). Finally, CFTR activity was inhibited by adding CFTR inhibitor-172 (inh-172, 10 µM) (Fig. 5A). For each epithelium, total CFTR activity was estimated as the amplitude of the current drop after addition of inh-172. Epithelia treated for 24 h with vehicle alone (DMSO) displayed a relatively small CFTR-mediated current (Fig. 5). Treatment with VX-661/VX-445 caused a significant increase in total CFTR current compared to vehicle-treated cells, while TAK-243 alone was not effective (Fig. 5). However, when epithelia were treated with the double-corrector combination in the presence of TAK-243, we observed a further increase in CFTR-dependent activity, with 100 nM TAK-243 being the most effective concentration and resulting in a 1.5-fold increase in total CFTR-mediated current (Fig. 5). The same results were consistently found on epithelia derived from two different F508del homozygous patients (Fig. 5).

**Fig. 6:**
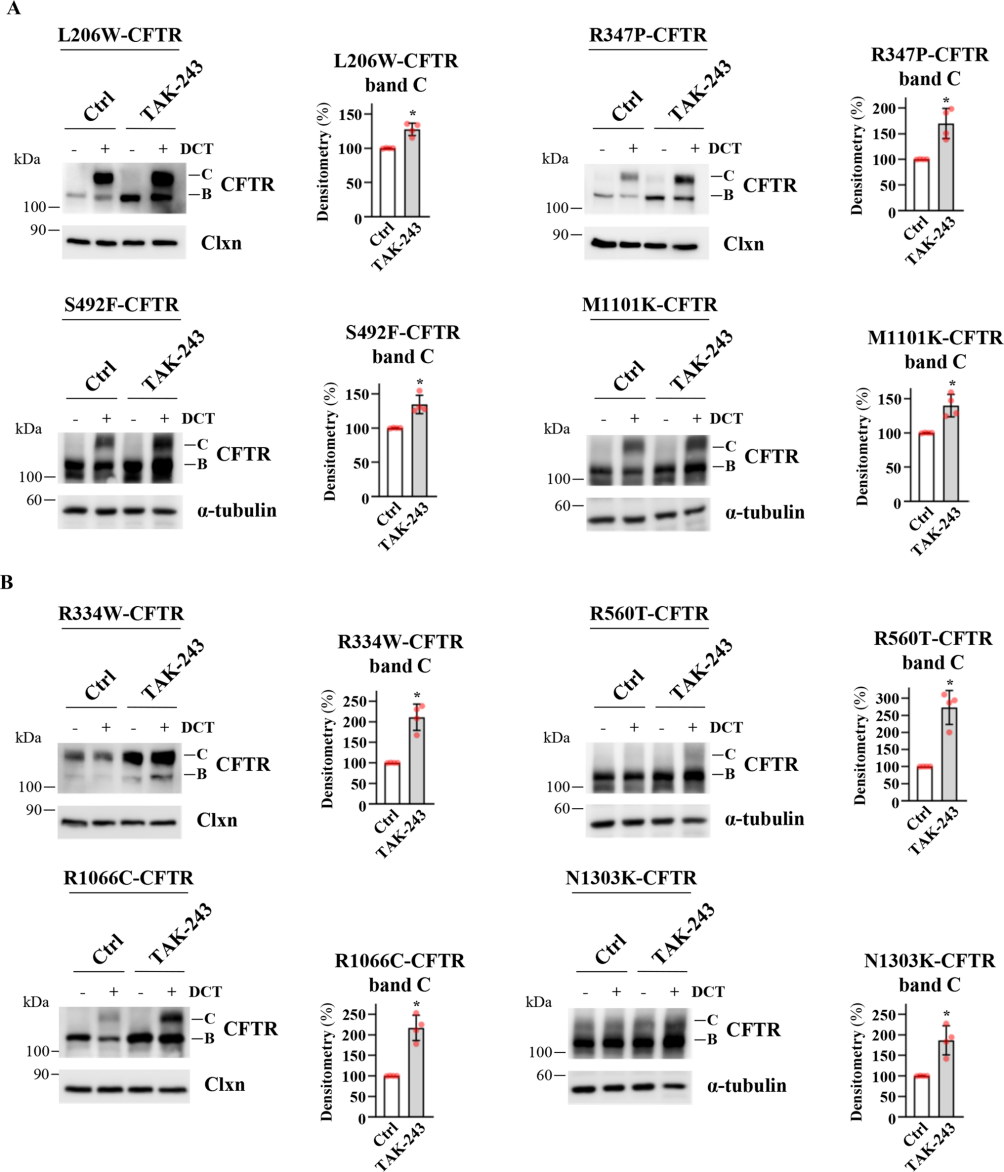
Effect of TAK-243 in combination or not with double-corrector treatment on the recovery of different CFTR pathologic mutants. CFBE41o- cells were transfected with CFTR vectors carrying the following point mutations: L206W, R347P, S492F, or M1101K in (**A**), and R334W, R560T, R1066C, or N1303K in (**B**). 24 h post-transfection, cells were treated with DMSO (Ctrl) or TAK-243 (200 nM) in combination or not with double-corrector treatment (DCT = 10 μM VX-661 + 3 μM VX-445) for further 24 h. Subsequently, cells were lysed and analyzed by western blot with anti-CFTR antibody. α-tubulin was used as a loading control. The graphs in each panel show the densitometric quantification of CFTR band C mutants of cells treated with DCT in the presence or absence of TAK-243. The values are expressed as a percentage of the cells treated only with DCT (means ± SD values, *n* = 4; *p < 0.05 vs Ctrl)

TAK-243 improves F508del-CFTR rescue induced by VX-445 and VX-661 of rare misfolded CFTR mutants in CFBE41o- cells. We wondered whether the addition of TAK-243 is also effective in combination with VX-445/VX-661 correctors in treating CFTR rare mutants displaying a trafficking defect. We therefore transiently transfected eight different mutants into CFBE41o- cells, comparing the rescue induced by correctors alone or in combination with TAK-243. We considered four mutants (L206W, R347P, S492F, and M1101K) for which the FDA recently approved Trikafta treatment [10] (Fig. 6A) and four mutants (N1303K, R334W, R560T, R1066C) not included in that approval (Fig. 6B). Although the effect is variable according to the type of mutation, combined treatment with TAK-243 and DCT leads to improved correction for all mutants compared to DCT alone (Fig. 6A, B).

**Fig. 7:**
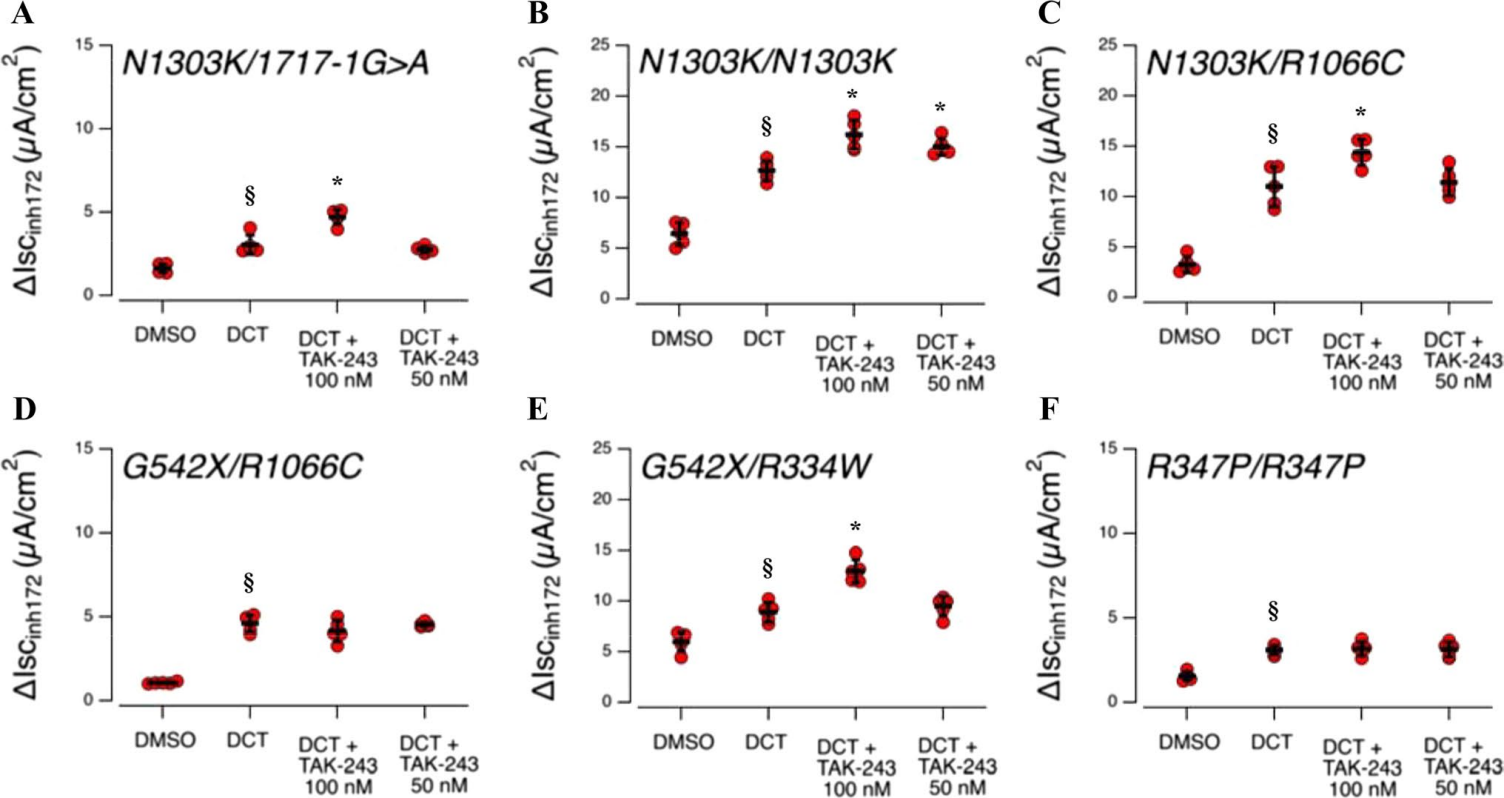
Functional evaluation of DCT and TAK-243 co-treatment on human nasal epithelial cells derived from CF patients bearing rare mutations. The graphs show the summaries of results obtained from short-circuit current recordings on nasal epithelia derived from different CF patients: **A** HNE011 (N1303K/1717-1G > A), **B** HNE084 (N1303K/N1303K), **C** HNE006 (N1303K/R1066C), **D** HNE001 (G542X/R1066C), **E** HNE016 (G542X/R334W), and **F** HNE020 (R347P/R347P). Data reported are the amplitude of the current blocked by 10 µM inh-172. Symbols indicate statistical significance of DCT vs. control (DMSO-treated): means ± SD values, ^§^*p* < 0.5; or of DCT plus TAK-243 vs DCT *p < 0.05

TAK-243 significantly increases CFTR conductance induced by elexacaftor/tezacaftor/ivacaftor in differentiated primary patient cells with N1303K and R334W mutations. For selected mutants (N1303K, R1066C, R334W, R347P), we further assayed the ability of TAK-243 to improve CFTR rescue induced by corrector treatment on well-differentiated primary cultures of airway epithelia. For this purpose, we used nasal cells derived from brushing. Nasal cells were expanded using a proliferative medium in the presence of SMAD inhibitors [27] and then conditionally reprogrammed to form fully differentiated nasal epithelia under ALI conditions. Nasal epithelia were then treated for 24 h with VX-445/VX-661 in the presence or absence of 100 nM of TAK-243 (the most effective compound concentration tested on F508del/F508del bronchial epithelia). Short-circuit current measurements were then taken to evaluate CFTR-dependent Cl- secretion (Fig. 7). After inhibition of ENaC using amiloride, CFTR activity was elicited by stimulation using the cAMP agonist followed by VX-770 (1 µM) plus apigenin as co-potentiator (25 µM) (only in the case of N1303K epithelia). Total CFTR-mediated current was then blocked by inh-172 (20 µM) (Fig. 7). In epithelia derived from an N1303K/1717-1G > A patient (HNE011), the only CFTR form that is expressed is N1303K-CFTR, since 1717-1G > A results in no CFTR mRNA expression [40]. Control epithelia (treated with DMSO alone) displayed a severely reduced total CFTR-mediated current (after stimulation using CPT-cAMP, VX-770, and apigenin). Treatment with VX-661/VX-445 caused a modest but significant increase in total CFTR current, which was further improved by co-treatment with TAK-243 (Fig. 7). Similar results were obtained when the compounds were tested on nasal epithelia derived from a patient homozygous for N1303K mutation (HNE084). In epithelia derived from an N1303K/R1066C patient (HNE006), both N1303K- and R1066C-CFTR are expressed. VX-661/VX-445 treatment caused a marked increase in CFTR-dependent Cl- secretion, which was further augmented when the epithelia were co-treated with correctors and TAK-243 (Fig. 7). To understand whether the increased activity was due to the rescue of both CFTR mutants, we examined the effect of the test compounds on epithelia derived from a G542X/R1066C (HNE001). CFTR activity in these cells was rescued by treatment with the two correctors, but no further effect was seen when TAK-243 was included in the treatment (Fig. 7). This result suggested that TAK-243 was effective only in rescuing N1303K-CFTR in HNE006 epithelia.

We also evaluated TAK-243’s ability to improve CFTR rescue using VX-661/VX-445 on two CFTR mutants—R347P and R334W—classified as class IV, namely displaying defective conductance. R347P mutant likely displays also a trafficking defect, as evidenced by previous work [41] and by the fact that it was included in the list of mutants for which Trikafta was approved. Conversely, R334W was considered to have a pure activity defect [42] based on heterologous expression system studies. However, recent data from the “Organoid Study R334W” clinical trial (NCT04254705) suggest that R334W might also be responsive to double-corrector treatment.

Thus, nasal epithelia derived from a G542X/R334W patient (HNE016) were treated for 24 h with DMSO alone or with VX-445/VX-661 in the presence or absence of 100 nM of TAK-243. Double-corrector treatment caused a significant increase in total CFTR-mediated current, which was further augmented by co-treatment with TAK-243 (Fig. 7). In nasal epithelia derived from an R347P patient (HNE020), CFTR activity was markedly reduced, which is consistent with the severity of the R347P activity defect. Treatment with VX-445/VX-661 caused a significant rescue of CFTR activity, although this was very modest. No further improvement was observed following co-treatment with TAK-243 (Fig. 7).

## Discussion

Increasing F508del-CFTR stability by both correcting mutant CFTR misfolding and modulating the cellular quality control system could be a successful strategy for targeted therapeutic intervention in CF. However, despite a large number of investigations, a drug targeting the ubiquitin cascade has not yet been incorporated into current pharmacological treatments for CF.

One possible explanation is that, as discussed above, most research efforts have been directed towards the identification and inhibition of the E3 ligases involved in the excessive polyubiquitination and premature degradation of the CFTR mutants. Unfortunately, several E3 enzymes are involved in this process, suggesting that the inhibition of multiple enzymes would be necessary to obtain the desired therapeutic result. We should also consider the possibility that multiple E3 targeting might prove to be insufficient due to either redundancy or compensatory mechanisms between E3 ligases [43]. To overcome these limitations, one option is to prevent the ubiquitination of F508del by taking action at the beginning of the ubiquitination cascade, as first suggested by Brodsky’s group [20, 22].

Here, we are investigating the potential therapeutic efficacy of a first-in-class UBA1 inhibitor [23], TAK-243, which acts upstream in the ubiquitination cascade and should therefore be more effective in preventing CFTR mutant degradation.

Our results show that TAK-243 treatment has a substantial effect on F508del-CFTR cell protein levels in CFBE41o- cells, inducing a dose-dependent increase in F508del-CFTR band B—the ER-resident protein—but without inducing the production of the fully glycosylated and plasma-membrane-resident band C (Fig. 1). Accordingly, TAK-243 treatment fails to induce a functional recovery of F508del-CFTR (Fig. 1).

This results might surprise, because in the presence of a larger amount of immature CFTR (band B), a consequent increase of mature species (band C) is expected as the maturation process should follow the mass action law. However, from what is known from previous studies [20, 36], our compound could be assimilated to a subset of “band B correctors” which might stabilize a yet partially misfolded protein that cannot be processed further (in the absence of other correctors), thus resulting in no mature CFTR expression and function.

The TAK-243 effect is related to its ability to increase the stability of F508del-CFTR, thus preventing its ubiquitination. Our results demonstrate that the inhibition of the E1 enzyme UBA1 is the main factor responsible for the effects observed, while the UBA6 and NAE enzymes—two secondary targets of TAK-243—are not involved in the control of F508del-CFTR stability. The lack of any effect by the NAE inhibitor Pevonedistat on F508del-CFTR stability was unexpected. NAE processes NEDD8 for binding to target substrates, and it has been shown previously that siRNA downregulation of NEDD8 induces a partial rescue of F508del-CFTR by reducing its ubiquitination [31]. However, our results clearly show that Pevonedistat, despite its efficacy in inhibiting cullin neddylation—the best-characterized reporter of NAE activity—is ineffective on F508del-CFTR maturation either alone or in combination with correctors, thus calling into question the role of neddylation in F508del-CFTR proteostasis regulation. The discrepancy between our results and the earlier findings is not likely to be caused by the use of different cellular models. Indeed, NEDD8 downregulation also induces F508del rescue in CFBE cells [31]. Rather, the discrepancy could be explained by the different target and approaches: RNA interference targeting NEDD8 in the previous study [31], and an enzymatic inhibitor targeting NAE in this study. By inhibiting NAE, we prevented protein neddylation but not NEDD8 protein interactions with specific binding partners.

As the increase in band B expression induced by TAK-243 is per se not sufficient to induce a functional rescue of the protein, we wondered whether this large amount of ER/Golgi-resident protein could be corrected and targeted to the plasma membrane. For this purpose, we combined the effect of TAK-243 with the two correctors (VX-445 and VX-661) included in the Trikafta therapy. Our results show that TAK-243 boosts the effects of VX-445/VX-661 on F508del-CFTR maturation not only in CFBE41o- cells (Fig. 2) but also in differentiated human epithelia from two different patients (Fig. 5), demonstrating that the extent of the channel rescue induced by correctors is closely linked to the cellular abundance of the protein.

Comparing the effect of TAK-243 with two different inhibitors of the proteasome (MG132 and VLX1570) (Fig. 3A), we also confirmed that an increasing amount of band B protein obtained by preventing F508del-CFTR proteasomal degradation is not useful from a therapeutic point of view [3, 44]. Only by acting upstream—preventing ubiquitination—can we provide a larger amount of protein that leads to increased channel rescue in the presence of correctors.

Available pharmacological approaches for treatment of F508del-CFTR patients target trafficking defects (VX-445 and VX-661) and gating defects (VX-770), whereas a molecule able to prevent protein ubiquitination and thus increase the protein amount is still absent in ongoing therapy and could represent a challenge for the future. In this paper, we have shown that TAK-243 used to target the quality control machinery of CFTR mutants both at ER and the plasma membrane could be the missing piece of the puzzle in combined CF pharmacological treatment.

However, we should bear in mind that an increase in band B production could be achieved not only by increasing the stability of the protein but also by inducing an increase in F508del-CFTR transcription, for example by miRNA targeting [45] or through the use of amplifiers [46, 47]. The final outcome is the same: more protein to be corrected and an improved functional channel recovery.

The proposed pharmacological treatment is not limited to F508del-CFTR, but could also be extended to other CFTR mutants. Indeed, we have assayed the effects of TAK-243 on eight different mutants (displaying defective maturation) expressed in CFBE41o- cells. TAK-243 used in combination with VX-445/VX-661 correctors is always effective in increasing channel rescue (Fig. 6). More importantly, this effect is also visible with mutants for which Trikafta is not approved, such as N1303K, the second most frequent class II CFTR mutation, as well as R560T and R334W. Moreover, using primary nasal epithelia derived from patients with different genotypes, we confirmed that TAK-243 is also effective in improving the rescue of R334W and, more importantly, of N1303K. For this latter mutant, however, TAK-243 does not ameliorate its severe gating defect, requiring stimulation with two different potentiators. Our results open the way for a possible improvement in the treatment of people with these mutations (Fig. 7).

However, the results obtained in CFBE41o- could not be replicated for all mutants when assayed in primary epithelia from patient cells. Indeed, TAK-243 did not show any improvement in chloride conductance when tested on epithelia derived from patients with R1066C and R347P mutations (Fig. 7). This result once again shows how the cellular environment is crucial in predicting the response to a molecule, and that promising results obtained with CFBE41o- cells are not a guarantee of compound efficacy and should also be reproduced on patients’ cells [10].

Regarding the possible use of TAK-243 in the pharmacological treatment of CF patients, although our promising results show its efficacy on primary airway epithelia, supporting its possible progression into clinical practice, the potential toxicity of this molecule must be carefully considered, since this treatment can prevent the entire ubiquitin cascade. However, our experiments show that to obtain the therapeutic effects, it is not necessary to achieve a complete or almost complete inhibition of UBA1 that would lead to the blocking of the entire ubiquitination machinery. Indeed, chronic low doses of TAK-243 are tolerated by CFBE41o- cell cultures, apparently without inducing any defects in proliferation and signs of endoplasmic reticulum stress (Fig. 4E, F). Moreover, we have shown that our chronic low-dose treatment is sufficient to increase the stabilization of the protein and amplify the effects of the correctors (Fig. 4A). Therefore, our data admit of the possibility that specific inhibitors of UBA1 could be considered in light of an improvement in the current therapeutic approach. Again, to reduce potential adverse effects, further studies will be necessary to investigate the feasibility of an aerosol treatment targeting only airway epithelia, since lung degeneration is the major cause of mortality in CF patients [1].

### Supplementary Information

Below is the link to the electronic supplementary material.

Supplementary file1: Fig. S1. (A) The viability of F508del-CFTR expressing CFBE41o- cells was assessed by the MTT assay after 24 h treatment with increasing concentrations (25, 50, 100, 200, or 400 nM) of TAK-243 and expressed as a percentage of control (means ± SD values, n = 5 (A), n = 3 (B); *p < 0.05 vs control). (B) The viability of F508del-CFTR expressing CFBE41o- cells was assessed by the MTT assay after 24 h treatment with increasing concentrations of Pevonedistat after 24 and 48 h treatment with increasing concentrations (0.1, 0.2, 0.4, 1, 5 µM for 24 h; 0.1, 0.2, 0.4, 1 µM for 48 h) and expressed as a percentage of control. (means ± SD values, n = 5 (A), n = 3 (B); *p < 0.05 vs control). (C) Immunoblot analysis of CFTR and NEDD8-Cullin in whole lysates from F508del-CFTR expressing CFBE41o- cells treated with DMSO (Ctrl) or increasing concentrations of Pevonedistat for 48 h. α-tubulin was used as loading control (n = 3) (left panel). Assessment of F508del-CFTR activity was carried out by HS-YFP assay in F508del-CFTR expressing CFBE41o- cells treated as indicated above or with double corrector treatment (DCT = 10 μM VX-661 + 3 μM VX-445) for 24 h. Centre panel exhibits representative traces measuring YFP quenching (n = 4), right panel shows the CFTR activity as a percentage of control (Scr) (means ± SD values, n = 8; *p < 0.05 vs Ctrl). (D) CFTR mRNA level determined by quantitative real-time PCR in F508del-CFTR expressing CFBE41o- cells were treated with DMSO (Ctrl) or TAK-243 (200 nM) for 24 h. CFTR mRNA expression was normalized to 18S RNA and reported relative to its expression in Ctrl cells that was arbitrarily set to 1 (means ± SD values, n = 5). (E) F508del-CFTR expressing CFBE41o- cells were treated with DMSO or 200 nM TAK-243 for 24 h, lysed, and analyzed by western blot with anti-CFTR antibody. Calnexin (Clxn) was used as a loading control. The lysates were used for the immunoprecipitation experiments of Fig. 2D. Fig. S2. (A) Immunoblot analysis of CFTR in whole lysates from F508del-CFTR expressing CFBE41o- cells treated with increasing concentrations of TAK-243 (50, 100, 200 nM) in combination with double corrector treatment (DCT = 10 μM VX-661 + 3 μM VX-445) for 24 h. Calnexin (Clxn) was used as a loading control. The figure panel is representative of four independent experiments. The lower panel shows the densitometric quantification of the immunostained F508del-CFTR band C. The values for CFTR band C are expressed as a percentage of the control cells (means ± SD values, n = 4; *p < 0.05 vs Ctrl). (B) F508del-CFTR expressing CFBE41o- cells were transfected with non-specific siRNA (Scr), or two different UBA1 specific siRNAs. After 24 h post-transfection cells were treated with DCT (10 μM VX-661 + 3 μM VX-445) for further 24 and lysed. Lysate proteins were analyzed by western blot with the indicated antibodies. Calnexin (Clxn) was used as a loading control. The figure panel is representative of three independent experiments. (C) Assessment of F508del-CFTR activity was carried out by HS-YFP assay in F508del-CFTR expressing CFBE41o- cells treated with DMSO (Ctrl) or TAK-243 (200 nM) or MG132 (1 μM) or VLX1570 (250 nM) in combination ( +) or not (-) with double corrector treatment (DCT = 10 μM VX-661 + 3 μM VX-445) for 24 h. Left panels exhibit representative traces measuring YFP quenching, right panels show the CFTR activity as a percentage of control cells not treated with DCT (Ctrl) (means ± SD values, n = 7; *p < 0.05 vs Ctrl, #p < 0.05 vs Ctrl with DCT). Fig. S3. (A) 300 µg of lysate proteins from F508del-CFTR expressing CFBE41o- cells untreated (Ctrl) or grew for at least 1 month in presence of TAK-243 (10 nM) were immunoprecipitated with a control antibody from the same class (Ctrl) or anti-CFTR (CFTR) antibody. The immunocomplexes were analyzed by western blot with the indicated antibodies (left panel). The figure panel is representative of four independent experiments. A long exposition of CFTR detection is also shown (l.e.: long exposition). The central panels show representative density profiles of CFTR and ubiquitin in the CFTR-immunoprecipitated samples. Quantification of the density profiles was performed in the right panels by integrating the profile curves in the indicated intervals of molecular weight (Ubiquitin: 220–350 kDa; Ub-CFTR: 220–350 kDa; CFTR band B: 130–150 kDa) (means ± SD; n = 4; *p < 0.05 vs Ctrl). (B) Nuclear staining (Hoechst) of F508del-CFTR expressing CFBE41o- cells chronically treated (at least 1 month) with DMSO (Ctrl) or TAK-243 (10 nM) magnification 20 × . (C) Cell cycle analysis by flow cytometry of F508del-CFTR expressing CFBE41o- cells chronically treated (at least 1 month) with DMSO (Ctrl) or TAK-243 (10 nM). On the right histogram data of the cell cycle analysis (means ± SD values, n = 3). (D) Densitometric quantification of the immunoblots of Fig. 4E. The values are expressed as a percentage of the control cells (dashed line) (means ± SD values, n = 4; *p < 0.05 vs Ctrl; #p < 0.05 vs chronic treated cells). (PDF 615 KB)

## Data Availability

The data that support the findings discussed here are available from the corresponding authors upon reasonable request.
