# Supplementary material for: Targeting the E1 ubiquitin-activating enzyme (UBA1) improves elexacaftor/tezacaftor/ivacaftor efficacy towards F508del and rare misfolded CFTR mutants
Source: Cell Mol Life Sci. 2022 Mar 16;79(4):192. doi: 10.1007/s00018-022-04215-3 (PMC8924136; doi:10.1007/s00018-022-04215-3)
Supplement: Supplementary file 1 — Supplementary file1: Fig. S1. (A) The viability of F508del-CFTR expressing CFBE41o- cells was assessed by the MTT assay after 24 h treatment with increasing concentrations (25, 50, 100, 200, or 400 nM) of TAK-243 and expressed as a percentage of control (means ± SD values, n = 5 (A), n = 3 (B); *p < 0.05 vs control). (B) The viability of F508del-CFTR expressing CFBE41o- cells was assessed by the MTT assay after 24 h treatment with increasing concentrations of Pevonedistat after 24 and 48 h treatment with increasing concentrations (0.1, 0.2, 0.4, 1, 5 µM for 24 h; 0.1, 0.2, 0.4, 1 µM for 48 h) and expressed as a percentage of control. (means ± SD values, n = 5 (A), n = 3 (B); *p < 0.05 vs control). (C) Immunoblot analysis of CFTR and NEDD8-Cullin in whole lysates from F508del-CFTR expressing CFBE41o- cells treated with DMSO (Ctrl) or increasing concentrations of Pevonedistat for 48 h. α-tubulin was used as loading control (n = 3) (left panel). Assessment of F508del-CFTR activity was carried out by HS-YFP assay in F508del-CFTR expressing CFBE41o- cells treated as indicated above or with double corrector treatment (DCT = 10 μM VX-661 + 3 μM VX-445) for 24 h. Centre panel exhibits representative traces measuring YFP quenching (n = 4), right panel shows the CFTR activity as a percentage of control (Scr) (means ± SD values, n = 8; *p < 0.05 vs Ctrl). (D) CFTR mRNA level determined by quantitative real-time PCR in F508del-CFTR expressing CFBE41o- cells were treated with DMSO (Ctrl) or TAK-243 (200 nM) for 24 h. CFTR mRNA expression was normalized to 18S RNA and reported relative to its expression in Ctrl cells that was arbitrarily set to 1 (means ± SD values, n = 5). (E) F508del-CFTR expressing CFBE41o- cells were treated with DMSO or 200 nM TAK-243 for 24 h, lysed, and analyzed by western blot with anti-CFTR antibody. Calnexin (Clxn) was used as a loading control. The lysates were used for the immunoprecipitation experiments of Fig. 2D. Fig. S2. (A) Immunoblot analysis of [file 18_2022_4215_MOESM1_ESM.pdf]

Figure S1

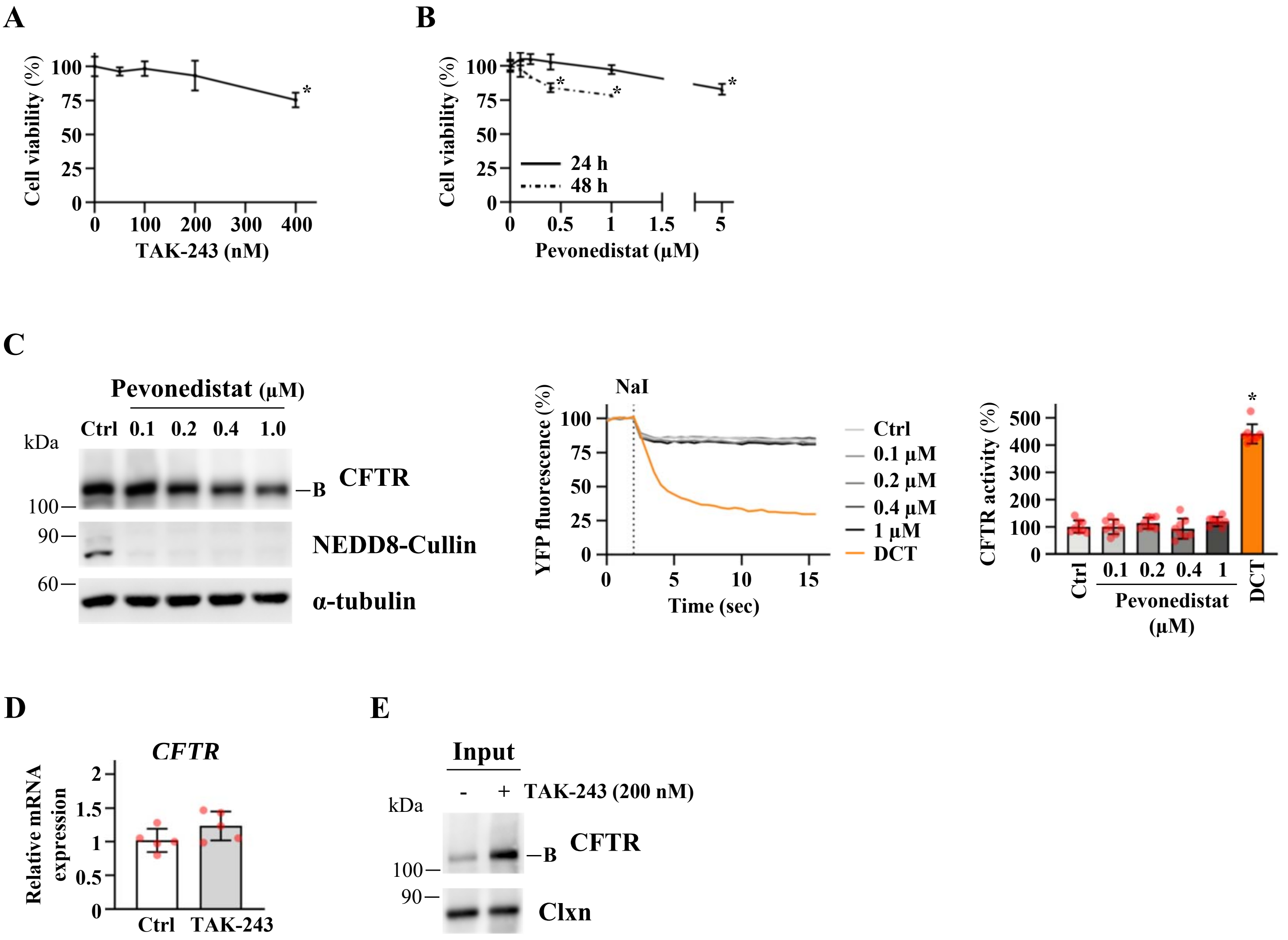

A
Cell viability (%)
TAK-243 (nM)
B
Cell viability (%)
24 h
48 h
Pevonedistat (µM)
C
Pevonedistat (µM)
Ctrl 0.1 0.2 0.4 1.0
kDa
100
90
60
B
CFTR
NEDD8-Cullin
α-tubulin
NaI
YFP fluorescence (%)
Time (sec)
Ctrl
0.1 µM
0.2 µM
0.4 µM
1 µM
DCT
CFTR activity (%)
Ctrl
0.1 0.2 0.4 1
Pevonedistat (µM)
DCT
D
CFTR
Relative mRNA expression
Ctrl
TAK-243
E
Input
- + TAK-243 (200 nM)
kDa
100
90
B
CFTR
Clxn

Fig. S1. (A) The viability of F508del-CFTR expressing CFBE41o- cells was assessed by the MTT assay after 24 h treatment with increasing concentrations (25, 50, 100, 200, or 400 nM) of TAK-243 and expressed as a percentage of control (means ± SD values, $n = 5$ (A), $n = 3$ (B); $^*p < 0.05$ *vs* control). **(B)** The viability of F508del-CFTR expressing CFBE41o- cells was assessed by the MTT assay after 24 h treatment with increasing concentrations of Pevonedistat after 24 and 48 h treatment with increasing concentrations (0.1, 0.2, 0.4, 1, 5 µM for 24 h; 0.1, 0.2, 0.4, 1 µM for 48 h) and expressed as a percentage of control. (means ± SD values, $n = 5$ (A), $n = 3$ (B); $^*p < 0.05$ *vs* control). **(C)** Immunoblot analysis of CFTR and NEDD8-Cullin in whole lysates from F508del-CFTR expressing CFBE41o- cells treated with DMSO (Ctrl) or increasing concentrations of Pevonedistat for 48 h. α-tubulin was used as loading control ($n = 3$) (left panel). Assessment of F508del-CFTR activity was carried out by HS-YFP assay in F508del-CFTR expressing CFBE41o- cells treated as indicated above or with double corrector treatment (DCT = 10 µM VX-661 + 3 µM VX-445) for 24 h. Centre panel exhibits representative traces measuring YFP quenching ($n = 4$), right panel shows the CFTR activity as a percentage of control (Scr) (means ± SD values, $n = 8$; $^*p < 0.05$ *vs* Ctrl). **(D)** *CFTR* mRNA level determined by quantitative real-time PCR in F508del-CFTR expressing CFBE41o- cells were treated with DMSO (Ctrl) or TAK-243 (200 nM) for 24 h. CFTR mRNA expression was normalized to 18S RNA and reported relative to its expression in Ctrl cells that was arbitrarily set to 1 (means ± SD values, $n = 5$). **(E)** F508del-CFTR expressing CFBE41o- cells were treated with DMSO or 200 nM TAK-243 for 24 h, lysed and analysed by western blot with anti-CFTR antibody. Calnexin (Clxn) was used as a loading control. The lysates were used for the immunoprecipitation experiments of Fig. 2D.

Figure S2

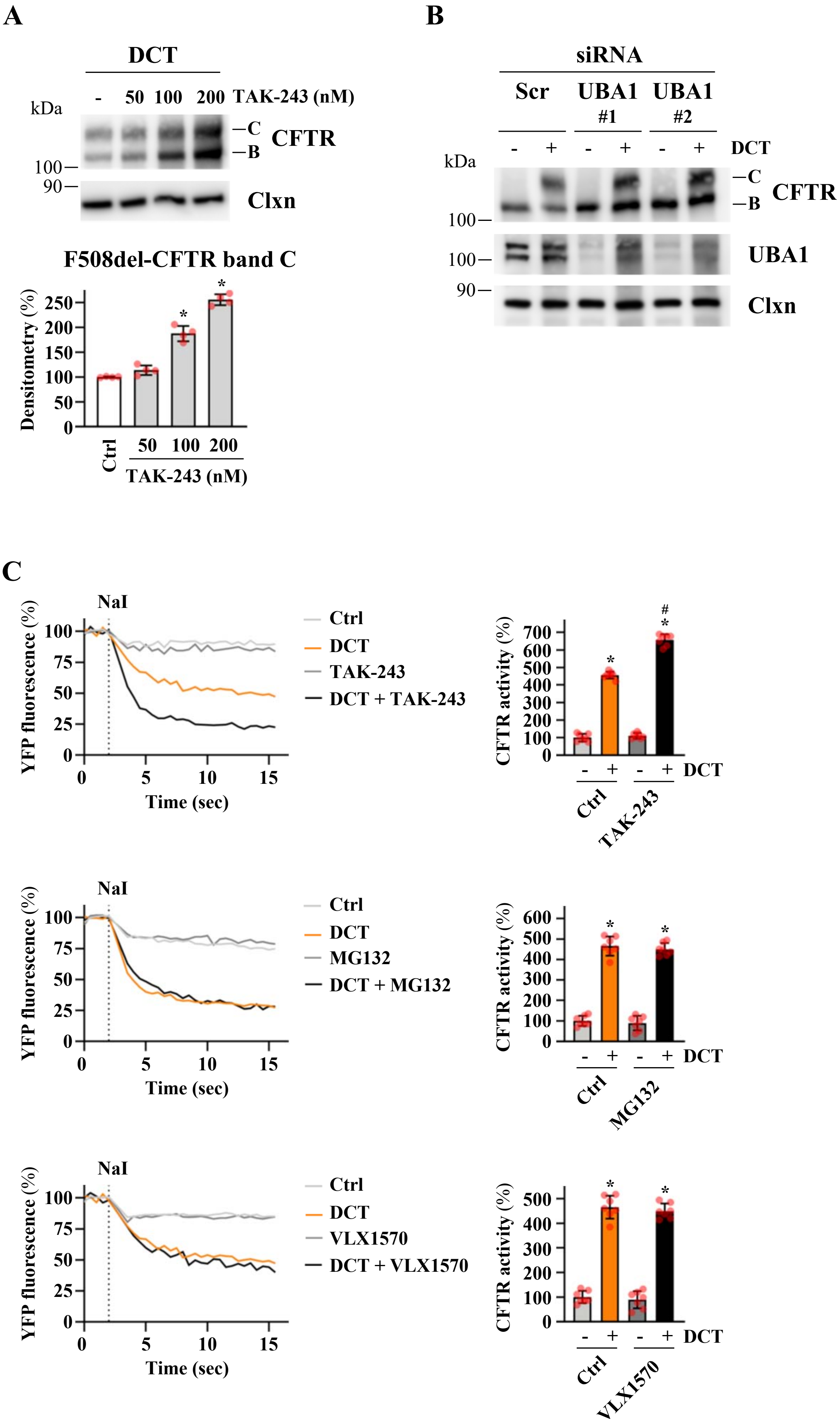
A
DCT
- 50 100 200 TAK-243 (nM)
kDa
C
B
CFTR
100
90
Clxn
F508del-CFTR band C
Densitometry (%)
Ctrl
50 100 200
TAK-243 (nM)
B
siRNA
Scr UBA1 #1 UBA1 #2
- + - + - + DCT
kDa
C
B
CFTR
100
100
UBA1
90
Clxn
C
NaI
YFP fluorescence (%)
Time (sec)
Ctrl
DCT
TAK-243
DCT + TAK-243
CFTR activity (%)
DCT
Ctrl
TAK-243
Ctrl
DCT
MG132
DCT + MG132
MG132
Ctrl
DCT
VLX1570
DCT + VLX1570
VLX1570

Fig. S2. (A) Immunoblot analysis of CFTR in whole lysates from F508del-CFTR expressing CFBE41o- cells treated with increasing concentrations of TAK-243 (50, 100, 200 nM) in combination with double corrector treatment (DCT = 10 μM VX-661 + 3 μM VX-445) for 24 h. Calnexin (Clxn) was used as a loading control. The figure panel is representative of four independent experiments. Lower panel shows the densitometric quantification of the immunostained F508del-CFTR band C. The values for CFTR band C are expressed as a percentage of the control cells (means ± SD values, $n = 4$; *$p < 0.05$ *vs* Ctrl). **(B)** F508del-CFTR expressing CFBE41o- cells were transfected with non-specific siRNA (Scr), or two different UBA1 specific siRNAs. After 24 h post-transfection cells were treated with DCT (10 μM VX-661 + 3 μM VX-445) for further 24 and lysed. Lysate proteins were analysed by western blot with the indicated antibodies. Calnexin (Clxn) was used as a loading control. The figure panel is representative of three independent experiments. **(C)** Assessment of F508del-CFTR activity was carried out by HS-YFP assay in F508del-CFTR expressing CFBE41o- cells treated with DMSO (Ctrl) or TAK-243 (200 nM) or MG132 (1 μM) or VLX1570 (250 nM) in combination (+) or not (-) with double corrector treatment (DCT = 10 μM VX-661 + 3 μM VX-445) for 24 h. Left panels exhibit representative traces measuring YFP quenching, right panels show the CFTR activity as a percentage of control cells not treated with DCT (Ctrl) (means ± SD values, $n = 7$; *$p < 0.05$ *vs* Ctrl, #$p < 0.05$ *vs* Ctrl with DCT).

Figure S3

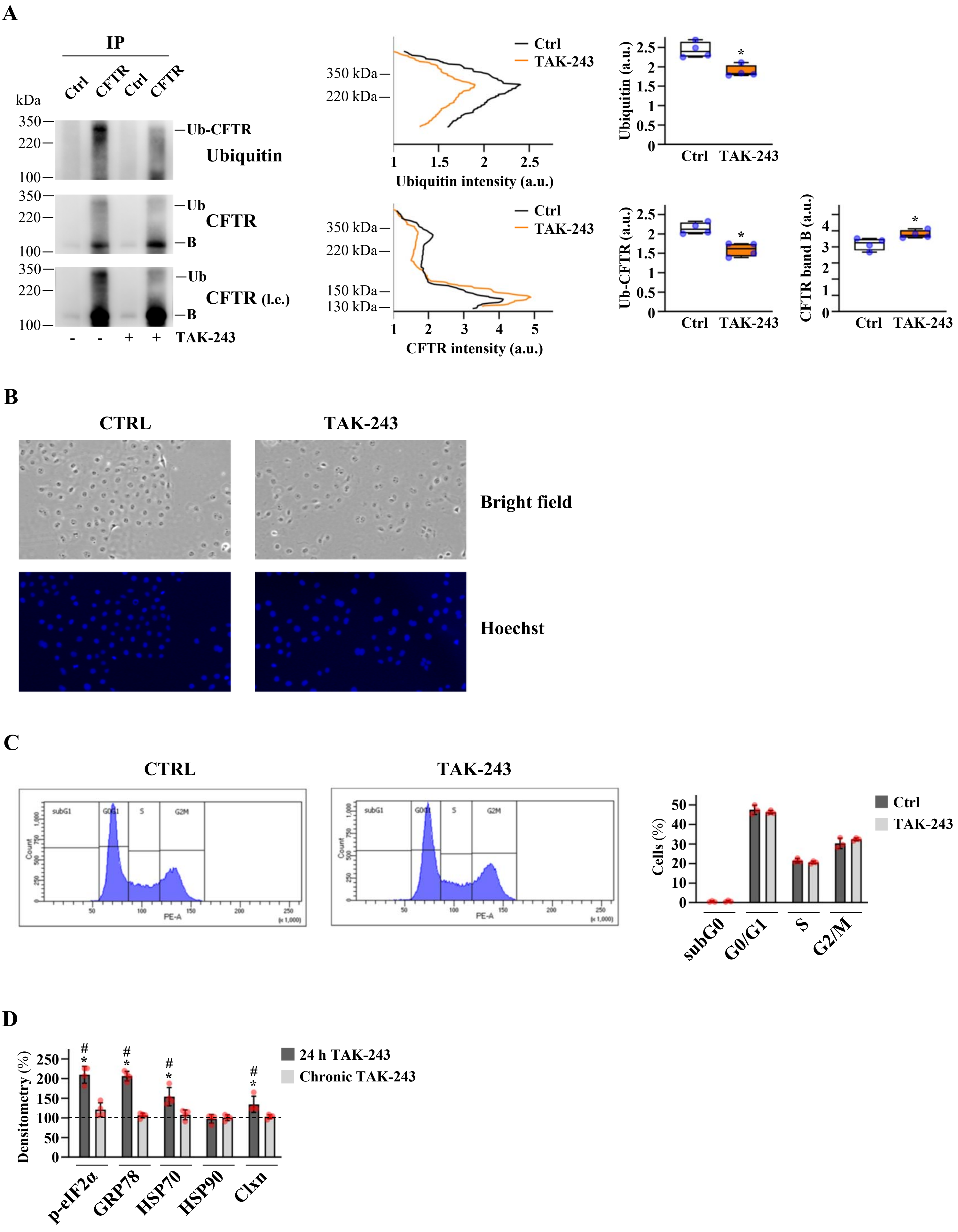

A
IP
Ctrl
CFTR
Ctrl
CFTR
kDa
350
220
100
Ub-CFTR
Ubiquitin
Ub
CFTR
B
CFTR (l.e.)
- - + + TAK-243
350 kDa
220 kDa
Ctrl
TAK-243
Ubiquitin intensity (a.u.)
150 kDa
130 kDa
CFTR intensity (a.u.)
Ubiquitin (a.u.)
Ub-CFTR (a.u.)
CFTR band B (a.u.)
B
CTRL
TAK-243
Bright field
Hoechst
C
CTRL
TAK-243
subG1
G0/G1
S
G2M
Count
PE-A
Cells (%)
Ctrl
TAK-243
subG0
G0/G1
S
G2/M
D
Densitometry (%)
24 h TAK-243
Chronic TAK-243
p-eIF2α
GRP78
HSP70
HSP90
Clxn

Fig. S3. (A) 300 µg of lysate proteins from F508del-CFTR expressing CFBE41o- cells untreated (Ctrl) or grew for at least 1 month in presence of TAK-243 (10 nM) were immunoprecipitated with a control antibody from the same class (Ctrl) or anti-CFTR (CFTR) antibody. The immunocomplexes were analysed by western blot with the indicated antibodies (left panel). The figure panel is representative of four independent experiments. A long exposition of CFTR detection is also shown (l.e.: long exposition). The central panels show representative density profiles of CFTR and ubiquitin in the CFTR-immunoprecipitated samples. Quantification of the density profiles was performed in the right panels by integrating the profile curves in the indicated intervals of molecular weight (Ubiquitin: 220-350 kDa; Ub-CFTR: 220-350 kDa; CFTR band B: 130-150 kDa) (means ± SD; n = 4; *p < 0.05 *vs* Ctrl). **(B)** Nuclear staining (Hoechst) of F508del-CFTR expressing CFBE41o- cells chronically treated (at least 1 months) with DMSO (Ctrl) or TAK-243 (10 nM) magnification 20×. **(C)** Cell cycle analysis analysis by flow cytometry of F508del-CFTR expressing CFBE41o- cells chronically treated (at least 1 months) with DMSO (Ctrl) or TAK-243 (10 nM). On the right histogram data of the cell cycle analysis (means ± SD values, n = 3). **(D)** Densitometric quantification of the immunoblots of Fig. 4E. The values are expressed as a percentage of the control cells (dashed line) (means ± SD values, n = 4; *p < 0.05 *vs* Ctrl; #p < 0.05 *vs* chronic treated cells).
